# Transcriptomic regulations of heat stress response in the liver of lactating dairy cows

**DOI:** 10.1186/s12864-023-09484-1

**Published:** 2023-07-20

**Authors:** Guangsheng Li, Xingtan Yu, Ananda B. Portela Fontoura, Awais Javaid, Víctor Sáinz de la Maza-Escolà, Nia S. Salandy, Susan L. Fubini, Ester Grilli, Joseph. W. McFadden, Jingyue Ellie Duan

**Affiliations:** 1 Department of Animal Science, College of Agriculture and Life Sciences, Cornell University, Ithaca, 14853 USA; 2 Dipartamento di Scienze Mediche Veterinarie, Università di Bologna, Bologna, 40064 Italy; 3 Department of Agriculture and Environmental Sciences, Tuskegee University, Tuskegee, 36088 USA; 4 Department of Clinical Sciences, College of Veterinary Medicine, Cornell University, Ithaca, 14853 USA; 5 VetAgro S.p.A, Reggio Emilia, 42124 Italy

**Keywords:** Dairy cow, Liver, Heat stress, Transcriptome, Metabolism

## Abstract

**Background:**

The global dairy industry is currently facing the challenge of heat stress (HS). Despite the implementation of various measures to mitigate the negative impact of HS on milk production, the cellular response of dairy cows to HS is still not well understood. Our study aims to analyze transcriptomic dynamics and functional changes in the liver of cows subjected to heat stress (HS). To achieve this, a total of 9 Holstein dairy cows were randomly selected from three environmental conditions - heat stress (HS), pair-fed (PF), and thermoneutral (TN) groups - and liver biopsies were obtained for transcriptome analysis.

**Results:**

Both the dry matter intake (DMI) and milk yield of cows in the HS group exhibited significant reduction compared to the TN group. Through liver transcriptomic analysis, 483 differentially expressed genes (DEGs) were identified among three experimental groups. Especially, we found all the protein coding genes in mitochondria were significantly downregulated under HS and 6 heat shock proteins were significant upregulated after HS exposure, indicating HS may affect mitochondria integrity and jeopardize the metabolic homeostasis in liver. Furthermore, Gene ontology (GO) enrichment of DEGs revealed that the protein folding pathway was upregulated while oxidative phosphorylation was downregulated in the HS group, corresponding to impaired energy production caused by mitochondria dysfunction.

**Conclusions:**

The liver transcriptome analysis generated a comprehensive gene expression regulation network upon HS in lactating dairy cows. Overall, this study provides novel insights into molecular and metabolic changes of cows conditioned under HS. The key genes and pathways identified in this study provided further understanding of transcriptome regulation of HS response and could serve as vital references to mitigate the HS effects on dairy cow health and productivity.

**Supplementary Information:**

The online version contains supplementary material available at 10.1186/s12864-023-09484-1.

## Introduction

Since global warming continues escalating together with relatively high ambient humidity levels in large confinement cow farms, heat stress (HS) has become a critical issue for the dairy industry [1]. In lactating dairy cows, the increased milk production can raise metabolic challenges to maintaining nutrient and energy homeostasis [1]. Especially during the hot days, when the internal heat load has already been elevated in cows to support high milk production, the excessive heat from the environment disrupts their metabolic balance [1, 2]. As a result, cows tend to be more susceptible to disease and have poorer reproductive performance under the HS condition [3, 4], and their inflammatory response and immune system are induced [5]. Importantly, overall milk production, including milk yield and quality, is dramatically reduced by HS in dairy cows [3, 4].

To understand the impact of HS on dairy cow homeostasis, tremendous efforts have been made to dissect the physiological response to heat stress in lactating cows. It is well-recognized that the respiratory rate and rectal temperature are elevated in heat-stressed cows, indicating a skewed thermal balance [6]. HS also reduces dry matter intake (DMI) in cows, posing a negative impact on nutrient transportation and energy partitioning [7]. In order to counteract the impact of reduced DMI, previous studies have employed the pair-feeding strategy, a feeding strategy matching the DMI of heat-stressed cows to thermal-neutral cows, and they have reported that the reduced DMI only accounts for around half of the milk reduction, indicating other mechanisms that have direct effects on the production performance in heat stressed cows [8, 9].

High throughput RNA sequencing (RNA-seq) technology allows capturing of the whole transcriptome dynamics in different tissues, making it possible to identify key genes or regulatory networks in HS responses of dairy cows. Recent studies have revealed that in the mammary gland of dairy cows, HS induced the upregulation of heat shock proteins (HSPs), while genes associated with milk protein synthesis were downregulated, including *CSN1S1*, *CSN2*, *STAT5A*, and *JAK2* [10]. Moreover, genes and pathways related to cell death, cytoskeleton degradation, and immune response were upregulated during the dry period in dairy cows under HS [11]. During the lactation period, genes related to amino acid and glucose transport such as *SLC38A10* and *SLC2A1* were downregulated. Inflammatory pathway involving NF-𝜿B and metabolic pathway involving PPAR𝛄 were activated and repressed by HS, respectively [7]. These studies suggest a direct impact of HS on the mammary gland at the transcriptomic level.

The liver is the essential site responsible for the whole body’s metabolism, which plays essential roles in lactation via providing glucose and amino acids for milk component synthesis [12]. Basal glucose concentrations were lower in dairy cows under HS, and metabolic pathways related to oxidative phosphorylation were activated in the liver at the proteomic level, contributing to reduced milk yields and the alteration of milk composition [13–15]. Moreover, a qPCR study of genes associated with carbohydrate metabolism and inflammatory processes revealed that HS could affect hepatic gene expression, such as the upregulation of *PCK1*, *PDK4*, *HP* and *NFKB1* in cows under HS [16]. Similar results were illustrated in sheep that HS impaired the liver carbon metabolism, the PPAR signaling pathway, and vitamin digestion and absorption [17]. Previous research also reported the potential crosstalk between liver and mammary gland that could regulate metabolism, innate immunity, and proliferation in lactating cows based on transcriptome profiles of these two organs [18]. However, the transcriptomic impact of HS on the liver is still largely unknown. Thus, our objectives are to generate a comprehensive profile of transcriptomic changes in the liver of lactating cows under HS and to identify potential candidate regulatory genes associated with HS-related metabolic dysregulation in dairy cows.

## Materials and methods

### Animals and sample collection

All experiment procedures were approved by the Cornell University Institutional Animal Care and Use Committee (#2018 − 0110). Animals in the present study were randomly picked (n = 9) from a total of 46 Holstein dairy cows that were part of another experiment. The experimental details have been previously described in a recent publication [19]. The temperature-humidity index (THI) was calculated based on the equation reported by Kendall et al. [20]:

THI = (1.8 x Temperature + 32) – [(0.55–0.0055 x Humidity) x (1.8 x Temperature − 26)].

Briefly, animals were transported from the Cornell University Research Center (Dryden, NY) to Cornell University Large Animal Research and Teaching Unit (Ithaca, NY) and acclimated in thermoneutrality (22.2 ± 0.25 °C; 44.9 ± 0.05% relative humidity; 68 ± 0.32 THI) for 7 days. Next, cows were balanced according to lactation, days carrying a calf, and milk yield before being allocated into three environmental conditions for 14 days: thermoneutrality (TN; n = 3, average days in milk = 190), heat stress (HS; 6 am-6 pm 27–37 °C, THI = 74–82; 6 pm-6 am 27 °C, THI = 74; n = 3, average days in milk = 211) and thermoneutrality but pair-fed to match the feed intake of heat stressed cows (PF; n = 3, average days in milk = 226). Cows were fed twice daily at 7:30 am and 4:30 pm and also milked twice daily at 6 am and 4 pm. Along with dry matter intake (DMI) and milk yield, cows were monitored thrice daily for clinical signs of HS (rectal temperature, skin temperature, and respiration rate) during the study. On day 12 of the treatment, liver biopsies were performed as previously described by Rico et al. [21] using aseptic techniques. Briefly, hair was clipped surrounding the 11th intercostal space, and the incision site was sanitized with iodine scrub and anesthetized with lidocaine HCl (12 ml; Vedco, lnc., Saint Joseph, MO) delivered subcutaneously. Following the collection of tissue, the biopsy site was stapled and sprayed with antiseptic, while liver tissue obtained from the cows was immediately frozen in dry ice and then transported to the lab where samples were kept in -80 °C until further RNA extraction.

### Liver RNA extraction

Total RNA was isolated from each sample using TRIzol reagent (Invitrogen, Carlsbad, CA), Phasemaker™ tubes (Invitrogen), and RNeasy Mini Kit (Qiagen) according to the manufacturer’s instructions with minor modifications. Specifically, tissue samples were homogenized in TRIzol and lysates were transferred to Phasemaker™ tubes with chloroform for 20 min rotation at 4 °C. The aqueous phase was then transferred to the gDNA elimination column for 1 min rotation at room temperature. The eluted RNA was washed with cold 70% ethanol, transferred to the RNeasy mini spin column, and washed with buffer RPE and RW1 (RNeasy Mini Kit). Finally, RNAs were eluted in RNAse-free water and concentrations were determined by a NanoDrop 1000 spectrophotometer (Thermo Scientific, Waltham, MA).

### Library preparation and sequencing

RNA quality control (QC), library construction, and RNA sequencing were performed through the service provided by Novogene Inc. (Sacramento, CA, USA). Briefly, RNA quality was evaluated on Bioanalyzer 2100 (Agilent) and only samples passed the QC were proceeded with the downstream process. Next, mRNA was enriched from total RNA using poly-T oligo-attached magnetic beads. After fragmentation, the first strand cDNA was synthesized using random hexamer primers followed by the second strand cDNA synthesis. The library was ready after a series of subsequent steps, including end pair, A-tailing, adapter ligation, size selection, amplification, and purification. Finally, the library was checked with Qubit and real-time PCR for qualification and evaluated on a bioanalyzer for fragment size distribution. Quantified libraries were pooled and sequenced on Illumina platforms in paired-end mode (2 × 150 bp).

### RNA-seq analyses

The adaptor removal and quality control of the raw sequencing reads were carried out using fastp (v0.23.2) [22]. Reads with a percentage of low-quality base (quality score < 20) > 40% were removed. Reads with length < 30 bp or with too much Ns (> 5%) were also removed in this study. The cow reference genome (ARS-UCD 1.2) was downloaded from UCSC database, and the alignment of clean reads were performed with STAR (v2.7.9a) [23] allowing no more than 3 mismatches. The raw read counts for each gene were extracted using featureCounts (v2.0.3) [24], and the gene expression level was normalized by transcripts per million (TPM) using IsoEM2 [25]. Then the principal component analysis (PCA) was performed using the top 3,000 variable genes identified by DEseq2 [26] R package to predict the correlations of the samples.

For the identification of differentially expressed genes (DEGs), the raw read count matrix was imported into DEseq2 [26] R package. The pair-wise comparison was performed among three conditions and DEGs were selected. The selecting criteria of DEGs was: Fold Change (FC) >1.5 and FDR < 0.1. Additionally, an unsupervised hierarchical clustering was applied for the whole DEG list to highlight gene expression pattern from different groups, and the heatmap was generated with ComplexHeatmap [27] package in R. The gene ontology (GO) and Kyoto Encyclopedia of Genes and Genomes (KEGG) pathway [28] enrichment of upregulated and downregulated DEGs under different environmental conditions were then performed using clusterProfiler (v4.0.5) [29] in R (v4.1.1).

### Real-time quantitative PCR validation

Real-time quantitative PCR (RT-qPCR) was performed to validate the results of RNA-seq analysis. A total of nine genes (*ACACA*, *CRYAB*, *DIO1*, *GPC3*, *HSPA1A*, *HSPB1*, *MIOX*, *PRAP1*, *WFDC2*) that were more than 2-fold differentially expressed were randomly selected. Total RNA was extracted from each sample as described above and 1 ug RNA was used for reverse transcription using iScript cDNA Synthesis Kit (Bio-Rad, Hercules, CA) according to the manufacturer’s instructions. RT-qPCR was conducted using SsoAdvanced Universal SYBR Green Supermix (Bio-Rad) in a CFX384 Touch Real-Time PCR machine (Bio-Rad). The reaction cycle was as follows: one cycle at 95 °C for 30 s, 40 cycles at 95 °C for 10 s, and at 60 °C for 30 s, and melting curve analysis using the instrument’s default setting. The primers were designed by NCBI Primer-BLAST and the primer list was shown in Additional file 1: Table S1. Glyceraldehyde-3-phosphate dehydrogenase (*GAPDH*) was used as the internal control gene. The relative expression was calculated with 2^−ΔΔCT^ method [30]. To check the consistency between RNA-seq and RT-qPCR, we calculated the R square of a linear regression between these two results.

### Regulatory factor identification and protein-protein interaction

We identified transcription factors (TFs) and transcription cofactors in cattle using Animal Transcription Factor Database [31]. In addition, we used the Search Tool for the Retrieval of Interacting Genes (STRING, v11.5, https://string-db.org /) to construct the protein-protein interaction (PPI) networks for the DEGs from each pair-wise comparison, and proteins with interaction score < 0.4 were removed from our results. The PPI network was visualized using Cytoscape (v3.9.1, https://cytoscape.org/) to show core hub genes.

### Statistical analysis

For DMI, milk yield and clinical phenotypes measured during the experiment, summary statistics were obtained using R software (v4.1.1). The statistical differences between different conditions were calculated using the ANOVA function in R with the following model:

$${Y}_{ij}= \mu +{A}_{i} + {B}_{j}+{C}_{k}+ {A}_{i} \times {B}_{j} +{e}_{ij}$$

Where ${Y}_{ij}$ = phenotypic value; $\mu$ = population mean; ${A}_{i}$ = environmental conditions; ${B}_{j}$ = days of treatment; ${C}_{k}$ = random effect of cow; ${A}_{i} \times {B}_{j}$ = the interaction effect between treatment and day; ${e}_{ij}$ = residual error.

For relative expression with qPCR, we follow the 2^−ΔΔCT^ method [30] as following three steps:

$$\Delta Ct\left( {sample} \right){\text{ }} = Ct\left( {Target{\text{ }}gene} \right) - Ct\left( {GAPDH} \right)$$

$$\Delta \Delta Ct{\text{ }} = \Delta Ct\left( {calibrator{\text{ }}group} \right) - \Delta Ct\left( {other{\text{ }}group} \right)$$

Fold change from calibrator group $= {2^{ - \Delta \Delta Ct}}$

To check the gene expression pattern between RNA-seq and qPCR results, we performed the Person Correlation analysis and calculated R^2^ values in each animal group using R software (v4.1.1).

## Results

### Impact of heat stress on feed consumption, milk yield and clinical phenotypes

**Fig. 1:**
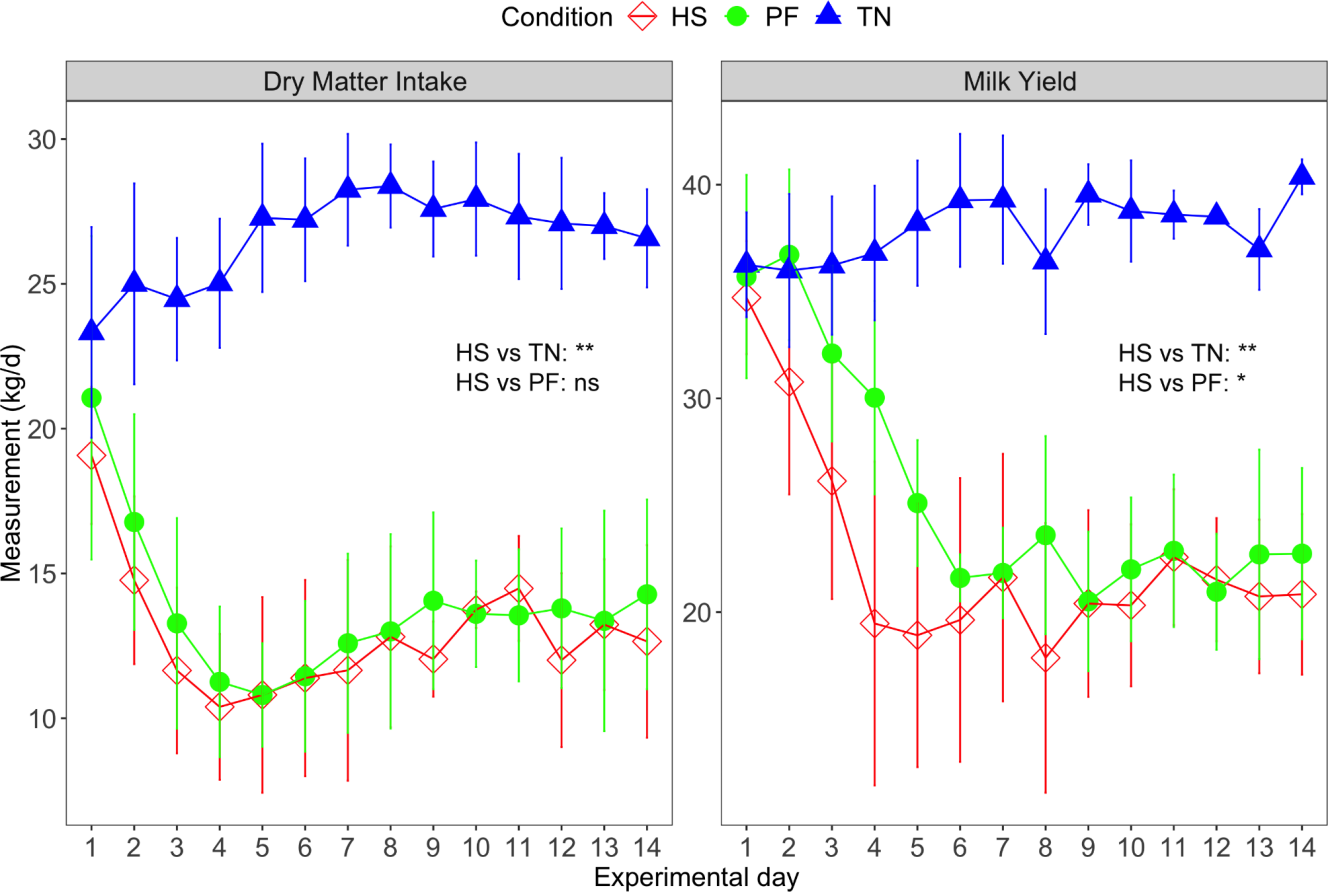
The dynamics of the dry matter intake and milk yield during the experiment. **: p < 0.05; *: p < 0.1; ns: no significance. The significance of the difference between different groups are calculated for the whole study period. The points show the mean value and the vertical lines show the standard error. TN: thermoneutrality; HS: heat stress; PF: pair-fed

During the experiment, the mean of daily DMI in the TN group fluctuated between 23.33 and 28.38 kg, which was significantly higher than that of the HS group (ANOVA test, *p* < 0.05, Fig. 1). To demonstrate that the reduced DMI in the HS group is partially causing milk yield reduction, we included a PF group that had a matched DMI to the low energy intake with the corresponding HS counterpart. As expected, the TN group exhibited a significantly higher milk production (37.94 ± 3.92 kg/d) than the HS or PF group (ANOVA test, *p* < 0.05, Fig. 1). Meanwhile, we observed a much faster rate of milk reduction in HS group compared to PF group in the first week, although the milk yield tended to be the same for the rest of experiment between these two groups (ANOVA test, p < 0.1, Fig. 1). For the clinical phenotypes, the HS group exhibited significantly higher levels of rectal temperature, skin temperature, and respiration rate compared to the PF or TN group (ANOVA test, p < 0.05, Additional file 4: Figure S1), while no clinical signs of HS were observed in the PF and TN groups.

### Summary statistics of the sequencing data

To identify how HS impacts cow liver function and define its role in milk yield reduction, we collected liver biopsy, performed RNA extraction, and analyzed transcriptome using RNA-seq. The Illumina PE150 platform was used to generate the high-throughput data, and the quality of sequencing data was summarized in Additional file 1: Table S2. A total of 399,528,794 raw reads were produced in the nine samples, and 397,164,236 clean reads passed the quality control, with an average error rate of 0.03%. The GC contents of the samples were within 46.39 − 49.44%, which was in consistent with base composition rules. The sequencing quality indicator (Q20 and Q30 $\ge$ 92.2%) suggested high sequencing quality in this study. For the alignment, the unique read mapping rate to the cow reference genome was around 95%, indicating that we had robust data quality in our bioinformatic analysis.

### PCA analysis and identification of differentially expressed genes

**Fig. 2:**
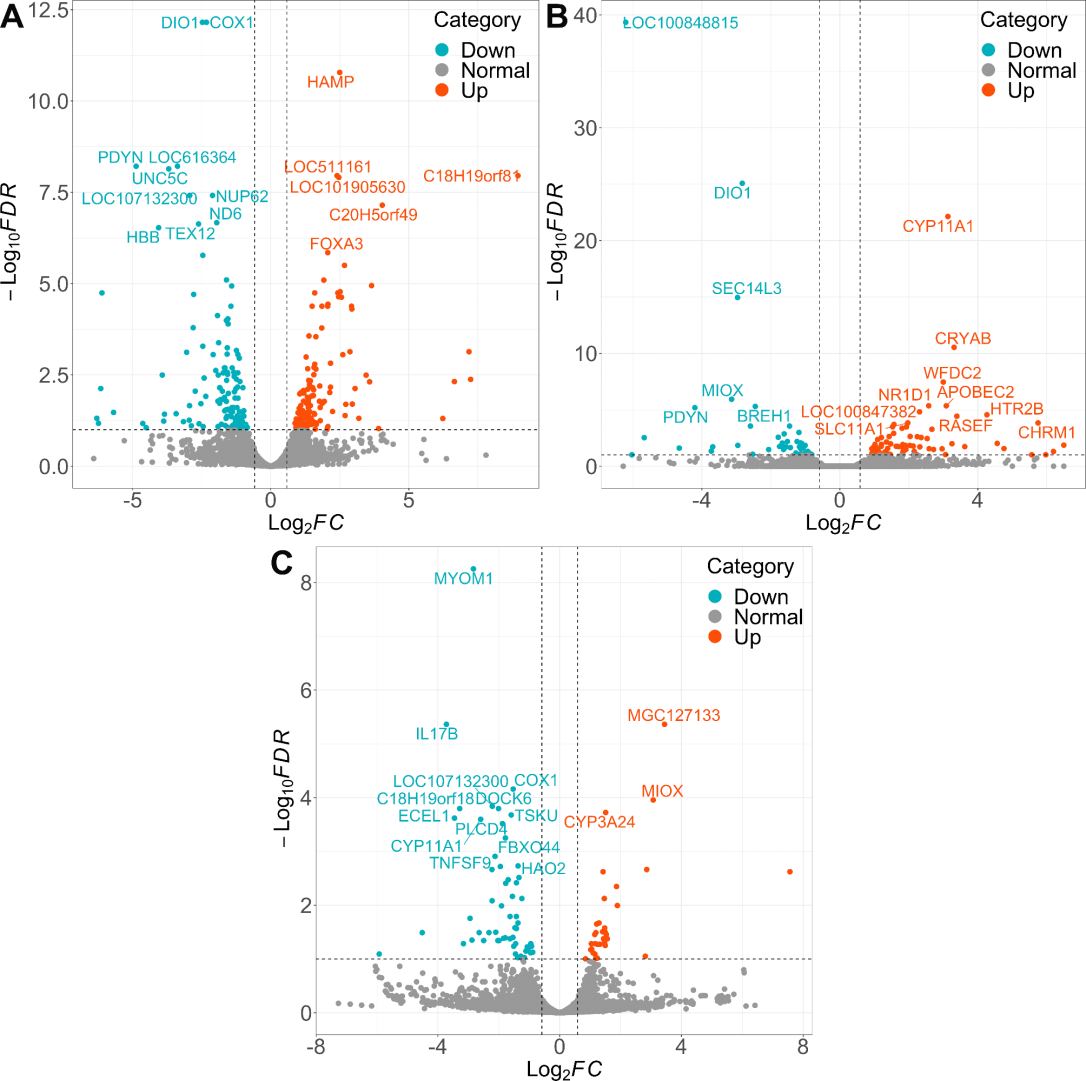
Distribution of DEGs in pairwise comparison. **A**: The DEGs of HS vs. TN;**B**: The DEGs of HS vs. PF; **C**: The DEGs of PF vs. TN. DEG: differentially expressed gene; TN: thermoneutrality; HS: heat stress; PF: pair-fed. The names of top 16 DEGs are annotated. Bule dots show the downregulated genes, red dots show the upregulated genes and gray dots mean not significant

To determine the correlation among the samples from different conditions, we conducted PCA analysis using the expression data of the top 3000 variable genes. The result revealed that all the samples formed three clusters, and these patterns confirmed that our RNA-seq data was consistent with our experimental design (Additional file 5: Figure S2). For the identification of transcript profile differences among TN, HS, and PF, we performed a pairwise comparison to obtain differentially expressed genes (DEGs). A total of 483 DEGs were identified in this study (Fig. 2A-C and Additional file 2: Table S3). The highest number of DEGs was found between the HS group and the TN group, with 133 genes in the HS group presenting upregulated expression, and 137 genes presenting downregulated expression (Table 1). Moreover, there were 122 and 91 DEGs in the comparisons between HS vs. PF and PF vs. TN respectively (Table 1). The overlaps of upregulated and downregulated genes among three comparisons were shown in Additional file 6: Figure S3. We found that there were 9 upregulated and 23 downregulated genes that were common in both the HS and PF groups, such as *GPC3*, *SDC1* and *UGP2*, suggesting these overlapping genes were regulated by reduced DMI during HS. Therefore, we removed these genes in further analysis when considering the direct effect of HS.

**Table 1:** Number of DEGs in pairwise comparison. DEG: differentially expressed gene; TN: thermoneutrality; HS: heat stress; PF: pair-fed; FC: fold change; FDR: false positive rate

Comparison	Total	Upregulation	Downregulation	Criteria
HS vs. TN	270	133	137	FC >1.5, FDR < 0.1
HS vs. PF	122	73	49	FC >1.5, FDR < 0.1
PF vs. TN	91	34	57	FC >1.5, FDR < 0.1

**Fig. 3:**
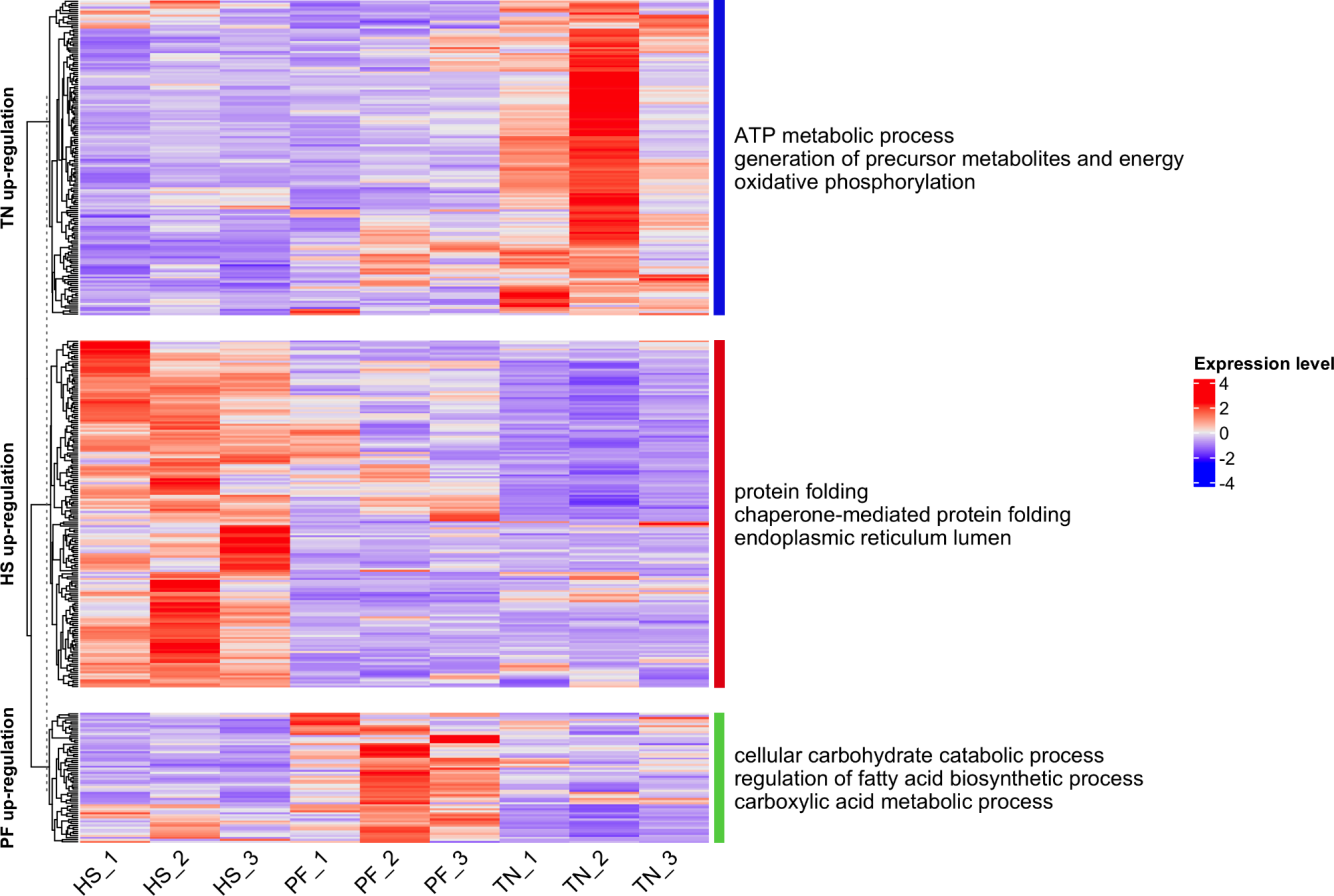
The heatmap of DEG expression levels. The X axis shows the samples, the Y axis shows three clusters, and the right labels show the GO enrichment annotation of the genes in each cluster. Each column stands for different samples and each row stands for different DEGs. The color indicates the TPM value, while red means high expression and blue means low expression. DEG: differentially expressed gene; TN: thermoneutrality; HS: heat stress; PF: pair-fed; TPM: transcripts per million; DEG: differentially expressed gene; GO: gene ontology

To obtain a deeper understanding of the gene expression patterns of the cows at different environmental conditions, we performed an unsupervised hierarchical clustering analysis of the whole DEG list (Fig. 3). We found that DEGs could be divided into three clusters, corresponding to their experimental groups with group-specific enrichment. Additionally, Gene ontology (GO) analysis showed that the genes in the TN-specific cluster were associated with ATP metabolic process and oxidative phosphorylation, and the genes in the HS-specific cluster could control chaperone-mediated protein folding and endoplasmic reticulum (ER) lumen function, while the genes in the PF-specific cluster were involved with cellular carbohydrate catabolic process and regulation of the fatty acid biosynthetic process.

### HS induced expression changes in heat shock proteins, mitochondrial and inflammatory related genes

**Fig. 4:**
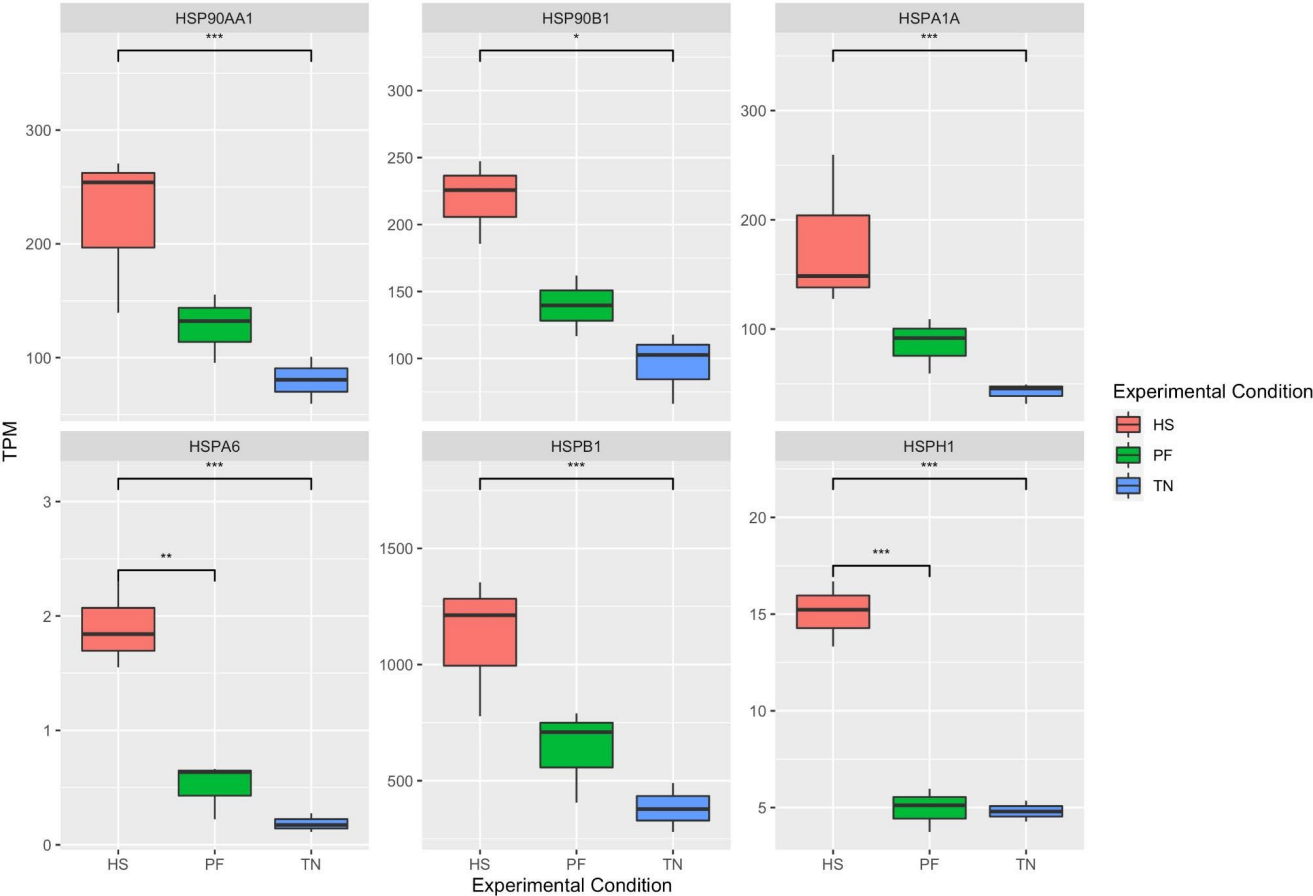
The expression pattern of differentially expressed HSPs. The X axis shows the three conditions, and Y axis shows TPM value. The boxplot shows the expression pattern of different HSPs, and the FDR value is obtained from DEseq2 result. HSP: heat shock protein; TN: thermoneutrality; HS: heat stress; PF: pair-fed; TPM: transcripts per million. ***: FDR < 0.01; **: FDR < 0.05; *: FDR < 0.1.

We next sought to evaluate the HS response in different functional categories, and performed a systematical analysis of genes related to heat shock proteins, mitochondria, and inflammation. In our study, there were 26 molecular chaperone-coding genes showing upregulation (FC > 1.5) in response to HS (Table 2). These included members of the heat shock protein (HSP) 90 family (*HSP90AA1*, *HSP90AB1*, *HSP90B1*), HSP40 family (*DNAJA1*, *DNAJA3*, *DNAJB1*, *DNAJB6*, *DNAJB9*, *DNAJC30*, *Sect. 63*), and HSP70 family (*HSPA1A*, *HSPA1L*, *HSPA5*, *HSPA6*, *HSPA8*, *HSPA9*, *HSPA13*). Moreover, in the comparison between the HS group and the TN group, 6 HSPs were significantly (FC > 1.5, FDR < 0.1) elevated in the HS group (Fig. 4). Two of the HSPs (*HSPA6* and *HSPH1*) were also the DEGs between the HS and PF group. The expression level of the HSPs was highlighted in Fig. 4. All of 6 HSP genes followed the same pattern: the significantly high expression in the HS group, lower expression in the PF group, and the lowest expression in the TN group, indicating chaperone-mediated protein folding activity was enhanced in the liver upon HS.

**Table 2:** List of upregulated chaperones in HS compared to TN. The upregulated chaperones are selected with a fold change > 1.5, and they are assigned to different HSP families. HSP: heat shock protein; TN: thermoneutrality; HS: heat stress

Category	Gene symbol	Entrez gene ID	Fold change (HS vs. TN)
Small HSPs	*HSPB1*	516,099	2.71062591
	*HSPB2*	508,671	2.05990198
	*HSPB3*	616,007	1.87241183
	*HSPB7*	512,251	1.87241183
	*CRYAB*	281,719	4.48700853
HSP40 gene family	*DNAJA1*	528,862	2.57980898
	*DNAJA3*	513,397	1.62626071
	*DNAJB1*	538,426	2.8248019
	*DNAJB6*	282,215	1.50182594
	*DNAJB9*	614,588	1.86674982
	*DNAJC30*	617,118	1.54695999
	*SEC63*	541,040	1.50926739
HSP70 gene family	*HSPA1A*	282,254	4.17746641
	*HSPA1L*	540,190	4.47467799
	*HSPA5*	415,113	1.79050575
	*HSPA6*	539,835	7.59592698
	*HSPA8*	281,831	1.51183354
	*HSPA9*	517,535	1.67380621
	*HSPA13*	505,907	1.53348317
HSP90 gene family	*HSP90AA1*	281,832	2.64569382
	*HSP90AB1*	767,874	1.86085867
	*HSP90B1*	282,646	2.1673705
Other chaperones	*ST13*	510,494	1.69790507
	*AHSA1*	539,220	2.03134865
	*PDIA3*	281,803	1.73705298
	*PDIA4*	415,110	3.01315146

**Fig. 5:**
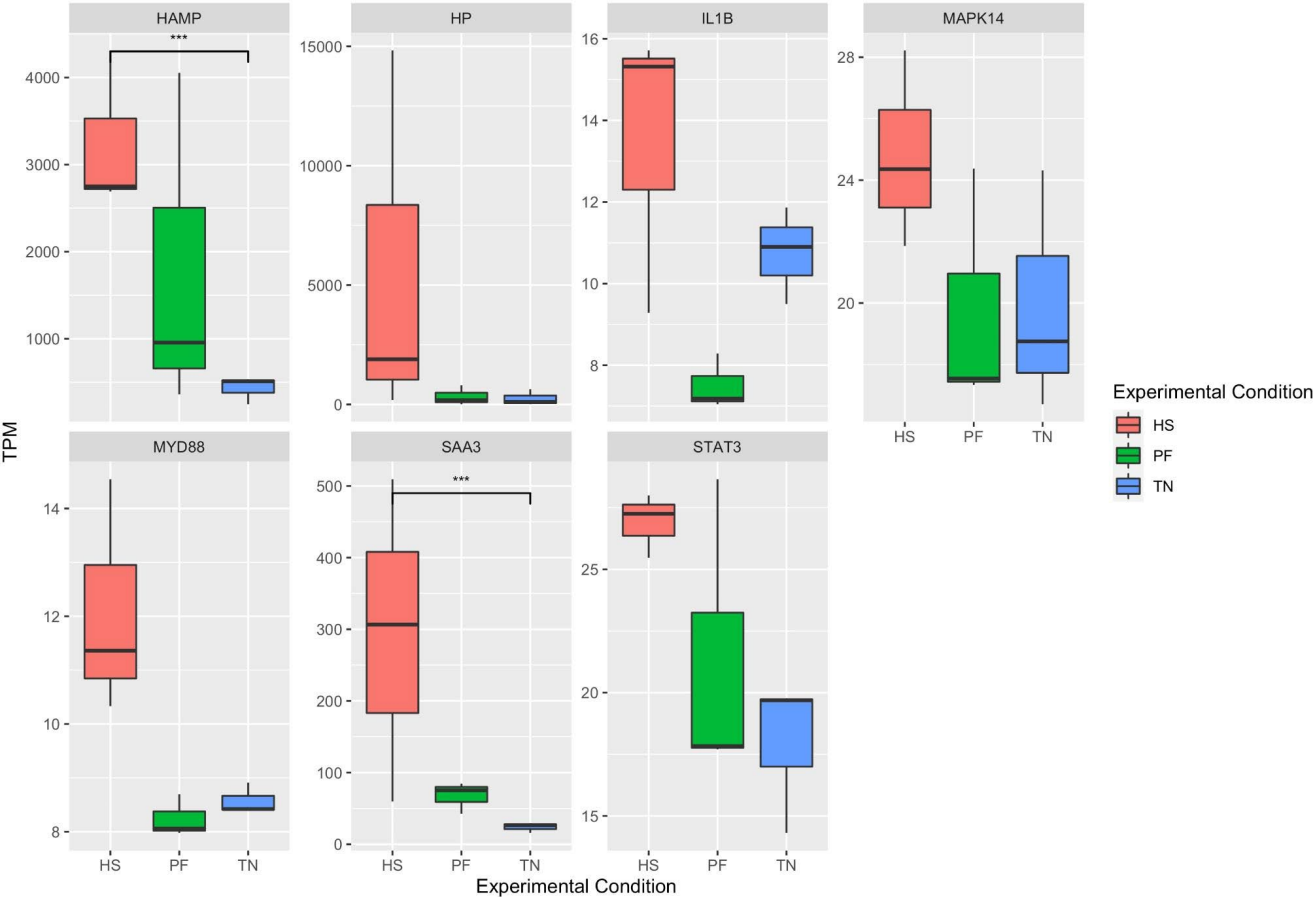
The expression pattern of liver inflammatory related genes. The X axis shows the three conditions, and Y axis shows TPM value. The boxplot shows the expression pattern of different genes, and the FDR value is obtained from DEseq2 result. TN: thermoneutrality; HS: heat stress; PF: pair-fed; TPM: transcripts per million. ***: FDR < 0.01; **: FDR < 0.05; *: FDR < 0.1.

Seven genes involved in liver immune response also elevated their expressions when exposed to HS compared to the TN (Fig. 5), with a fold change ranging from 1.2 to 22. Among them, *HAMP* and *SAA3* were significantly upregulated in the HS cows compared to the TN groups (DEseq2, FDR < 0.01), while no significant difference was found between the PF group and the TN group.

**Fig. 6:**
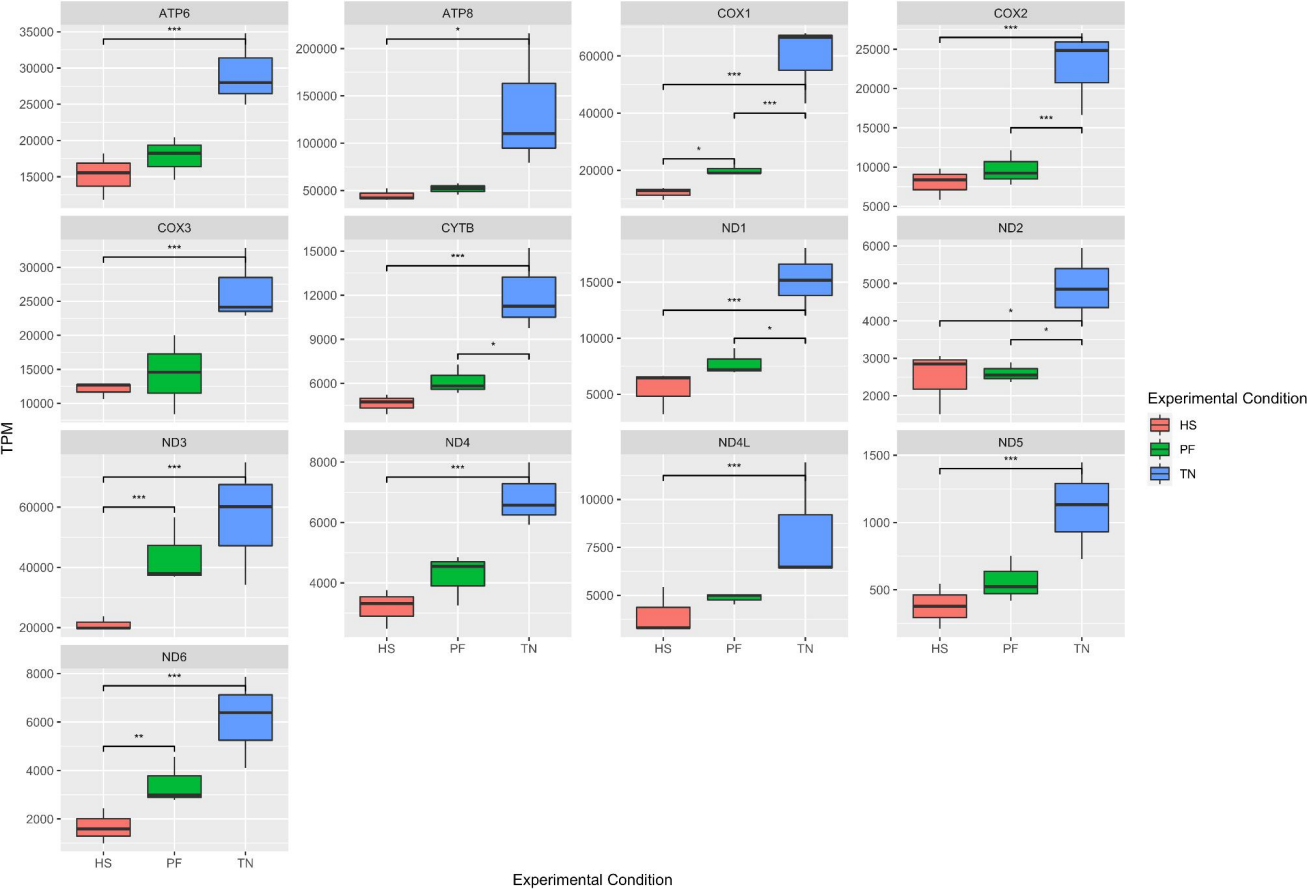
The expression pattern of mitochondrial genes. The X axis shows the three conditions, and Y axis shows TPM value. The boxplot shows the expression pattern of 13 protein coding genes in mitochondria, and the FDR value is obtained from DEseq2 result. TN: thermoneutrality; HS: heat stress; PF: pair-fed; TPM: transcripts per million. ***: FDR < 0.01; **: FDR < 0.05; *: FDR < 0.1.

Moreover, we found that all 13 protein-coding genes in mitochondria showed a significant (DEseq2, FDR < 0.01) downregulation in the HS group compared to the TN group (Fig. 6). Five of these mitochondrial genes were also the downregulated DEGs between the PF group and the TN group, including *COX1*, *COX2*, *CYTB*, *ND1* and *ND2*, while *COX1*, *ND3* and *ND6* were also significantly downregulated in the HS group compared to the PF group. The overall downregulation of mitochondrial genes implied that cellular energy generation was disrupted in the liver in both PF and HS, with a more server impact in the HS condition.

### Functional enrichment analysis of differentially expressed genes

**Fig. 7:**
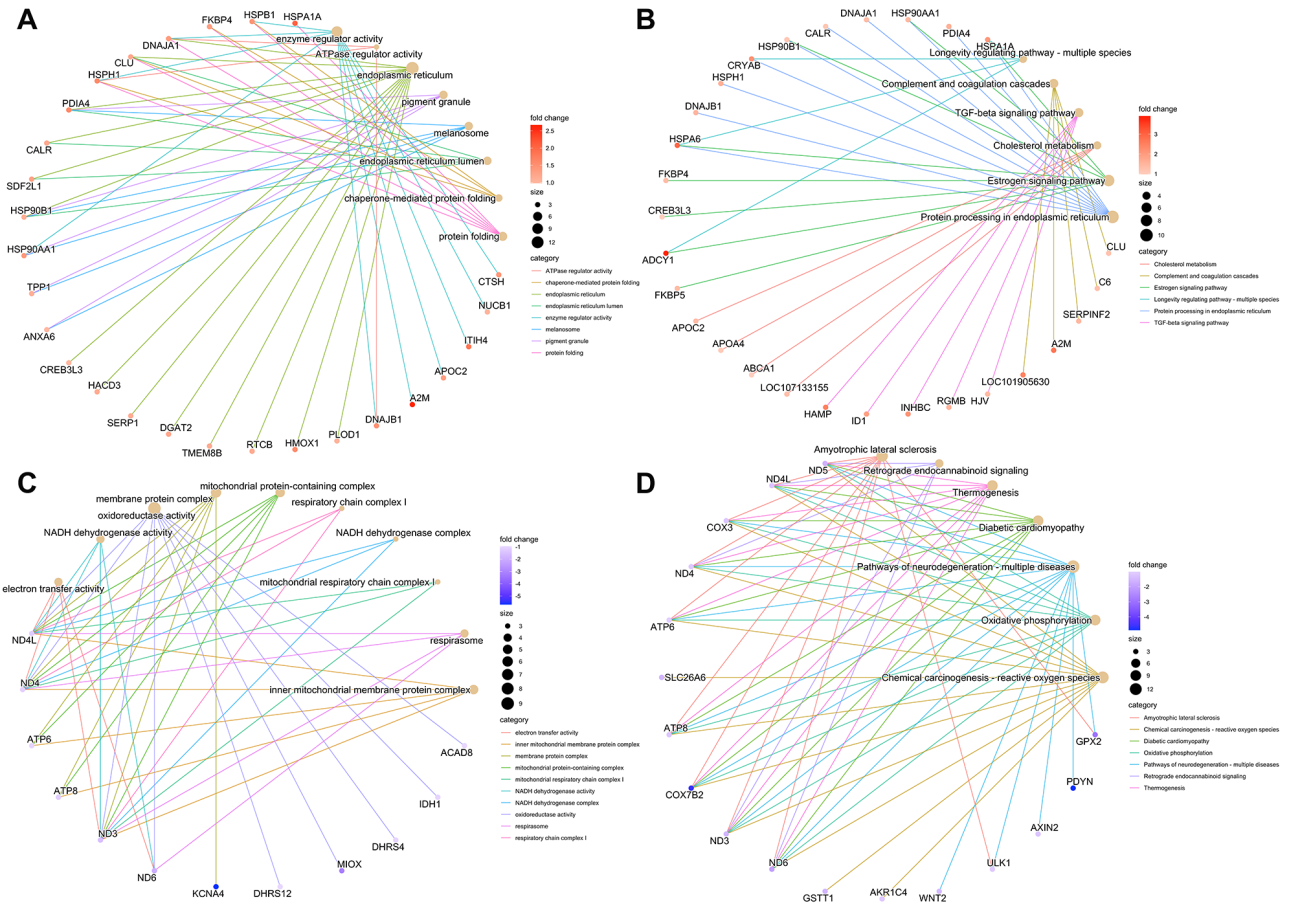
The enrichment results of DEGs. **A**: The GO enrichment of upregulated DEGs under HS; **B**: The KEGG enrichment of upregulated DEGs under HS; **C**: The GO enrichment of downregulated DEGs under HS; **D**: The KEGG enrichment of downregulated DEGs under HS. The brown node shows the enriched terms, and the size of brown node indicates the associated genes. The red or blue node shows the individual genes within each term, and the gradient of the gene color indicates the value of log2(Fold change). GO: gene ontology; KEGG: Kyoto Encyclopedia of Genes and Genomes; HS: heat stress; PF: pair-fed

Next, we explored how HS regulated gene expression and the corresponding functional pathway. To eliminate the effects triggered by energy deficit due to low DMI, we excluded the DEGs that overlapped with the PF condition. In total, 171 genes were upregulated, and 142 genes were downregulated under the HS condition. These genes were enriched in 29 GO terms, including 2 biological processes, 17 cellular components, and 10 molecular functions, and were involved with 36 KEGG pathways (Additional file 3: Table S4). The top GO term and KEGG pathway enrichments were presented in Fig. 7. Our results showed that enzyme regulator activity, ATPase regulator activity, and protein folding were upregulated, while inner mitochondrial membrane protein complex and electron transfer activity were downregulated under HS. Correspondingly, protein processing in ER, TGF-beta signaling, and cholesterol metabolism pathways were upregulated, while the oxidative phosphorylation pathway was downregulated in the heat-stressed cows.

Similarly, we also analyzed the GO term of DEGs between the PF group and the TN group. We observed that the cellular carbohydrate metabolic process and carboxylic acid metabolic process were upregulated, but oxidoreductase activity was downregulated in the PF group (Additional file 7: Figure S4). At the same time, pathway enrichment results indicated that ascorbate and aldarate metabolism was upregulated, while thermogenesis and cytokine-cytokine receptor interaction were downregulated for the PF cows.

### Protein-protein interaction (PPI) of differentially expressed genes

Next, we conducted protein interaction analysis to study the correlations among different genes. According to the DEG lists from pairwise comparisons, the PPI networks among the corresponding proteins of DEGs were obtained in this study (Additional file 8: Figure S5). Our results identified proteins that could be used as hub proteins and had multiple correlations with other proteins. In the HS group, the upregulated proteins with interactions greater than 10 were *APOA4*, *CDH1*, *AHSA1*, *CALR*, *DNAJA1*, *DNAJB1*, and major HSP family members, including *HSPA1A*, *HSP90AA1*, *HSP90B1*, *HSPH1*, and *HSPA6*. In the PF group, the upregulated proteins with more than three interactions were *PHGDH* and *LDHA*. Moreover, strong interconnections among 13 mitochondrial genes were observed both in the HS group and the PF group, and these genes formed a single cluster in our results (Additional file 8: Figure S5).

### Transcription factors, transcription cofactors and regulation network in response to heat stress

We delved deeper into the transcription regulation in the heat stress responses, by examining whether HS could change the expression of the TF genes. In this study, 18 transcription regulatory genes were found as DEGs in the comparison between HS and TN, including 10 TFs and 8 transcription cofactors (Table 3). Two upregulated members of the HSP family, *DNAJB1* and *HSPA1A*, could work as transcription cofactors in our study. Furthermore, these two genes were also identified as one of the hub proteins in our PPI networks.

**Table 3:** List of differentially expressed TFs and transcription cofactors. All the factors are selected from the DEGs between HS and TN group. DEG: differentially expressed gene; TF: transcription factor; TN: thermoneutrality; HS: heat stress

Category	Gene symbol	Entrez gene ID	Fold change (HS vs. TN)
	*RORC*	527,470	-2.6335949
	*ZNF653*	516,232	-2.1862458
Transcription factors	*ASCL1*	540,473	-2.185384
	*ZNF304*	526,123	-2.1060374
	*ATF5*	515,654	-1.8304779
	*CREB3L3*	513,010	1.98205159
	*ATOH8*	616,225	2.09380181
	*NR1D1*	768,225	2.73720746
	*ID1*	497,011	3.78636863
	*FOXA3*	503,622	4.18418466
Transcription cofactors	*KCNIP4*	614,299	-7.7435502
	*SGK1*	515,854	-1.9991956
	*CALR*	281,036	2.27455154
	*CLU*	280,750	2.33026672
	*WWC1*	520,730	2.39896652
	*DNAJB1*	538,426	2.8248019
	*HMOX1*	513,221	2.95082623
	*HSPA1A*	282,254	4.17746641

### Validation of RNA-seq by RT-qPCR

**Fig. 8:**
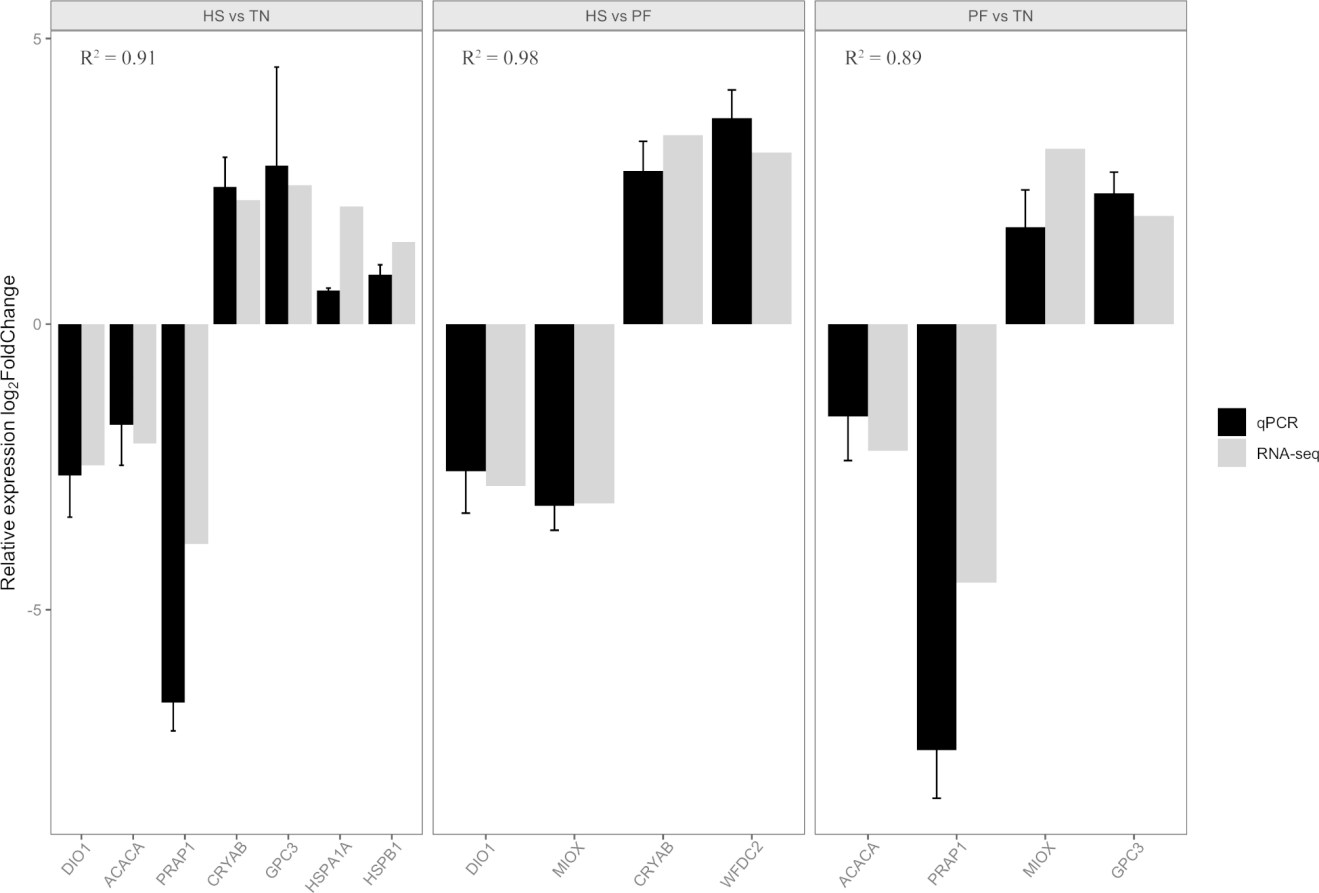
The validation result of RT-qPCR for pairwise comparisons. X axis shows the different genes, and Y axis shows relative expression log2FoldChange. R^2^ shows the linear regression coefficient, and the error bar stands for standard error. TN: thermoneutrality; HS: heat stress; PF: pair-fed

To validate the gene expression analysis derived from the RNA-seq, a total of 9 genes that were more than 2-fold differentially expressed were selected to compare with the relative expression level from RT-qPCR. We associated the gene expression fold change between RNA-seq and RT-qPCR in different conditions respectively (Fig. 8). High correlations (R^2^ ≥ 0.89) of gene expression fold change were found between these two methods, suggesting that our RNA-seq data was reliable and could capture the gene expression pattern accurately from the samples.

## Discussion

**Fig. 9:**
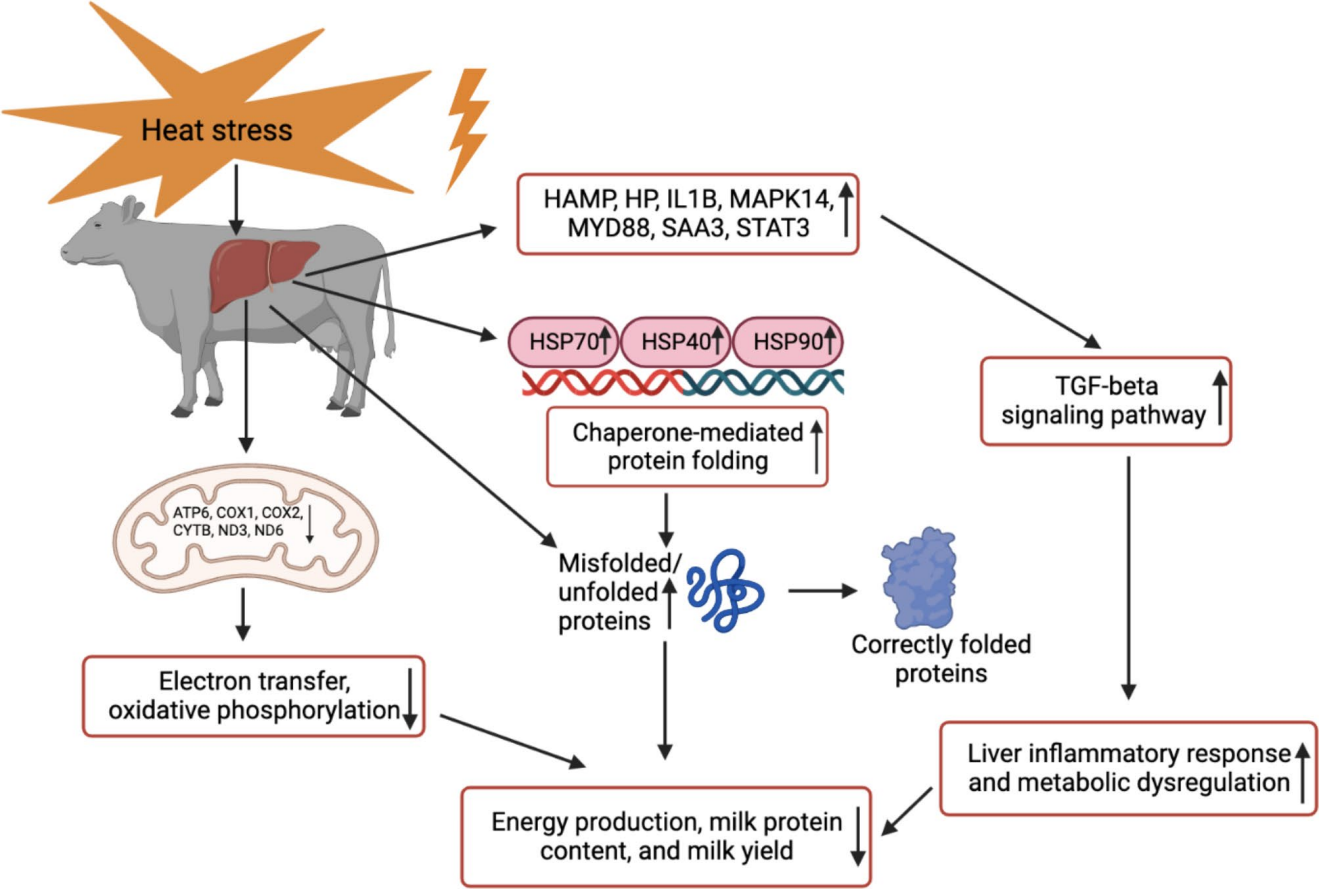
The liver response under HS in lactating dairy cows. Mitochondria dysfunction, upregulation of heat shock proteins and inflammatory signals can contribute to impaired production performance in cows under HS. HSP: heat shock protein; HS: heat stress

The central question we have is what mechanisms other than reduced DMI affect the production performance in heat-stressed cows. Based on all the findings described in detail above, here we summarized a liver regulation network in response to HS for cows (Fig. 9). It appears that HS directly affects liver homeostasis in three major aspects: (1) HS induces protein structures misfolding and elevates chaperone-mediated protein folding activities; (2) HS activates liver inflammatory responses and cellular cytokines to maintain essential biological processes; (3) HS damages the mitochondria membrane and perturbed mitochondrial function in energy generation by reducing oxidative phosphorylation. In total, energy production is shifted from milk protein synthesis to tackle the metabolic issues caused by HS, therefore the milk yield is significantly reduced in the cows. Overall, our work provided new insights into how HS impacts milk production by mechanisms of induced liver dysfunction.

### Effect of heat stress on production performance

Many factors of dairy animals, including reproduction, growth, and lactation, can be significantly affected by environmental factors such as HS. Elevated THI is detrimental to the maintenance of thermal equilibrium and leads to HS problems in the dairy industry [32]. In previous research, Johnson [33] found that milk yield and DMI declined significantly when the maximum THI reached 77. It is also well-documented that reduced DMI is a conserved response in heat-stressed animals and is a universal strategy to control metabolic heat production [6, 34, 35]. In this study, we were able to reach a THI of 82 for HS conditions during daytime and successfully observed the typical HS response in the cows, including the significant upregulation of body temperature and respiration rate, and the dramatic reduction of milk yield and DMI in the HS group compared to the TN group (Figure S1). As shown in Fig. 1, the overall DMI reduction of heat-stressed cows was around 50%, combined with a 40% reduction in milk yield. Importantly, according to the PF group, we calculated the expected milk production based on the DMI in the HS group. Then we performed a linear regression analysis between the expected and observed milk production in the HS group. The results showed that the declined DMI could explain about 70% of the change in milk yield, which was consistent with previous research [13, 36].

### Heat stress induced the upregulation of chaperones related to protein folding

It has been well recognized that in the cell, large proportions of newly synthesized proteins need assistance from molecular chaperones to reach their correct folding states efficiently [37]. Furthermore, molecular chaperones that are transcriptionally elevated under stress regulate the restoration of proteostasis through multiple aspects, such as preventing protein aggregation or facilitating refolding [38, 39].

As a major component of molecular chaperones, the HSP members have been significantly upregulated in our HS cows, including the HSP70 family, HSP40 family, and HSP90 family. Among HSPs, the HSP70 family is the highly conserved and the most abundant protein across organisms [40]. Upon heat shock, HSP70 is activated and removes the denatured or abnormal proteins in the cell, which could improve cell viability and its resistance to heat stress [41]. Furthermore, HSP70 had been reported to be the most active and vital regulator of thermal adaptation in livestock, and elevated HSP70 expression under HS could be found in dairy cows [42], buffalo [43], goats [44], and sheep [45]. The variant analyses revealed that a member of the HSP70 family (*HSPA6*) was the candidate gene for heat tolerance in Angus cattle [46], which also showed significant upregulation under HS in our data. Overall, our results exhibited a great level of consistency with HS study in other domestic species.

HSP40 is derived from the DNAJ protein family and works as an HSP70 cochaperone. HSP40 can balance the interaction between HSP70 and its unfolded substrates, and it also enhances the ATPase activity of HSP70 by a J domain [47]. Previous studies revealed that the HSP40 isoform DNAJB6 acted as potent suppressors of misfolded ploy-glutamine protein aggregation and cytotoxicity in vitro and in vivo, which was beneficial to the thermo-tolerance of cells [48]. Based on our results, two members of HSP40 (*DNAJA1* and *DNAJB1*) genes showed increased expression (DEseq2, FDR < 0.02) under HS compared to the TN group. Furthermore, the PPI network pointed out that HSP40 and HSP70 had a strong correlation with each other, and they expressed synergistically, indicating that HSP40 might play a critical role in cow liver response to HS by cochaperone misfolded protein to maintain the cellular proteostasis.

The HSP90 family is another necessary chaperone group downstream of HSP70, and it helps proteins to achieve ultimate structural maturation and conformational change to maintain homeostasis and cellular integrity under HS [44, 49]. In this study, two members of the HSP90 family (*HSP90AA1* and *HSP90B1*) genes were significantly upregulated (DEseq2, FDR < 0.1) in the HS group, similar elevation was also observed for *AHSA1*, which was an essential chaperone for activating the ATPase activity of HSP90 [50]. *AHSA1* cooperates with the middle domain of HSP90 and promotes target protein activation [51]. Based on these results, we proposed that HSP members were vital for correcting misfolded proteins and activating functional pathways under HS.

### Effect of heat stress on liver immune response and inflammation

The immune response is one of the mechanisms that evolved to counteract the side effects of environmental stressors and to improve both cellular and physiological adaptation in mammals [52]. HS is found to induce inflammatory and acute phase responses in pigs and dairy cows [53, 54]. Fontoura et al. [19] showed that HS could increase total tract gastrointestinal permeability in lactating cows, which might be detrimental to the natural barriers against bacteria. In our study, two inflammatory signaling genes, *MYD88* and *STAT3*, were activated in response to HS. It has been reported that *MYD88* cooperates with HSP60 to inhibit the apoptosis of B cells [55]. Previous studies also observed the expression of *MYD88* was significantly up-regulated in the spleen of yellow-feather broilers induced by HS [56]. *STAT3* is a member of the STAT family, and persistent activation of *STAT3* can be a hallmark of a variety of pathologies and underpins altered transcriptional responses [57]. Moreover, *STAT3* could assist Janus kinase 2 (JAK2) to mediate inflammatory responses caused by heatstroke in rats [58].

Moreover, the cow liver is the main site to produce acute phase proteins (APPs) in response to pro-inflammatory cytokines [59]. In our study, we also observed the activation of inflammatory mediators and APPs genes upon HS, including *HAMP*, *HP*, *IL1B*, *MAPK14*, and *SAA3*. *IL1B* is a typical cytokine produced by inflammation in cows, which plays an important role in improving intestinal permeability [60]. *HP* and *SAA3* are known markers of inflammation and they are also good indicators of cattle stress [61]. A previous study in pigs reported that HS-induced HP and *SAA3* synthesis in the liver [62], which was consistent with our results. For related pathways, these inflammatory genes are correlated with TGF-beta and MAPK signaling pathways in cows. Combined with all the findings, our study revealed that HS compromised the health status of lactating cows, specifically, the liver immune system was activated in response to HS.

### Effect of heat stress on mitochondrial function

Mitochondria is responsible for up to 90% of ATP consumption in different tissues [63]. Previous studies in cow liver showed that mitochondrial DNA copy numbers were higher in late lactation than in early lactation, indicating the elevated activities of mitochondria [64]. A recent study also reported that genes involved in mitochondrial biogenesis were upregulated in cow liver during lactation, indicating that adaptations in hepatic mitochondrial physiology were integral to supporting high milk production [65]. Furthermore, previous studies in rat liver found that HS could increase the permeability of the mitochondrial inner membrane and impair oxidative phosphorylation, leading to a dramatic reduction in ATP synthesis [66].

In this study, we observed that all 13 protein-coding genes in liver mitochondria were significantly downregulated under HS compared to the TN group, while only 5 of these genes showed significant downregulation in PF group compared to TN group. Furthermore, 3 of the mitochondrial genes also showed significantly low expression in HS group compared to PF group. According to the enrichment results of the mitochondrial genes that were exclusively impaired by HS, they can control electron transfer, NADH dehydrogenase, membrane protein complex, and respiratory chain. We noted that all these genes are associated with the oxidative phosphorylation pathway in cows, which has a critical function in liver energy production. Combined with the milk reduction we observed in the heat-stressed cows, we proposed that HS dysregulates the mitochondrial function in the cow liver and affects its ATP production process. Consequently, there is no sufficient energy to support milk component synthesis, which leads to a low milk yield. Moreover, the downregulation of all mitochondrial genes when exposed to HS could be a possible reason to explain the lower milk yield in the HS group than the PF group even if they were provided the same amount of DMI. However, further research is needed to profile how liver mitochondria damage contributes to lactation dysfunction in cows under HS.

### Differentially expressed transcription factors and transcription cofactors in response to heat stress

In previous studies, researchers found milk protein content was significantly reduced under HS, indicating that protein synthesis was disarranged [9, 19]. It has been reported that protein synthesis depends on the gene transcription level, which is regulated by TFs and transcription cofactors [67]. TFs bind directly to specific sequence elements involved with gene promoters and enhancers, while transcription cofactors are brought to promoters by TFs to either repress or enhance gene expression [67].

In this study, TFs and cofactors associated with ER stress and antiapoptotic process were upregulated in HS cows, including *ATOH8*, *CREB3L3*, *FOXA3*, *ID1*, *NR1D1*, *CALR*, *CLU*, and *HMOX1*. *ATOH8* can regulate plasma iron and bind to the promoter of *HAMP* to activate inflammatory signals [68]. *CREB3L3* is responsible for triglyceride and glucose metabolism, and acute phase response activation in the liver [69]. High expression of *FOXA3* is found to be induced by ER stress and can govern liver lipid synthetic genes directly [70]. *ID1*, *NR1D1*, *CALR*, *CLU*, and *HMOX1* are important regulators of cellular antioxidant response and can reduce cell apoptosis under HS [71–73]. Furthermore, we found *ASCL1* and *KCNIP4* were downregulated under HS. Both genes are strongly associated with the protein phosphorylation process and are considered key regulators of milk fat synthesis [74]. Taken together, these results indicated that the HS-induced transcription regulation activities were limited in increasing the basic cellular function for its survival and could not recover the side effect of metabolism turbulence under HS, which may contribute to the impaired milk production in lactating dairy cows.

## Conclusions

In this study, the effect of HS on lactating dairy cows was demonstrated through detailed liver transcriptome analysis using RNA-seq. Our data evaluated the gene expression pattern in different conditions, and the corresponding biological process and pathways affected by HS were identified. Several heat-responsive TFs and transcription cofactors were also found in our study. Furthermore, we defined three major liver response pathways that contribute to milk reduction under HS, including inflammatory signals, misfolded proteins, and mitochondrial dysregulation. The key genes, TFs, and cofactors identified in this study provided valuable reference to understand the genetic mechanism of HS response and could be useful candidates for selective breeding or nutritional or pharmacological target to improve thermal tolerance in dairy cows, which will be beneficial to the dairy industry and animal welfare.

## Electronic supplementary material

Below is the link to the electronic supplementary material.

Supplementary Material 1

Supplementary Material 2

Supplementary Material 3

Supplementary Material 4

Supplementary Material 5

Supplementary Material 6

Supplementary Material 7

Supplementary Material 8

Supplementary Material 9

## Data Availability

All data generated or analyzed during this study are deposited to NCBI GEO database (GSE226351).

## Abbreviations

HS: Heat stress
DMI: Dry matter intake
THI: Temperature-humidity index
RNA-seq: RNA sequencing
TN: Thermoneutrality
HS: Heat stress
PF: Pair-fed
PCA: Principal component analysis
TPM: Transcripts per million
DEG: Differentially expressed gene
GO: Gene ontology
KEGG: Kyoto Encyclopedia of Genes and Genomes
FC: Fold change
GAPDH: Glyceraldehyde-3-phosphate dehydrogenase
RT-qPCR: Real-time quantitative PCR
TF: Transcription factor
PPI: Protein-protein interaction
HSP: Heat shock protein
FDR: False positive rate

## References

[1] Chebel RC, Santos JE, Reynolds JP, Cerri RL, Juchem SO, Overton M (2004). Factors affecting conception rate after artificial insemination and pregnancy loss in lactating dairy cows. Anim Reprod Sci.

[2] Mader TL, Davis M, Brown-Brandl T (2006). Environmental factors influencing heat stress in feedlot cattle. J Anim Sci.

[3] Aguilar I, Misztal I, Tsuruta S (2010). Genetic trends of milk yield under heat stress for US Holsteins. J Dairy Sci.

[4] Nardone A, Ronchi B, Lacetera N, Ranieri MS, Bernabucci U (2010). Effects of climate changes on animal production and sustainability of livestock systems. Livest Sci.

[5] Yadav B, Singh G, Wankar A, Dutta N, Chaturvedi VB, Verma MR (2016). Effect of simulated heat stress on Digestibility, Methane Emission and metabolic adaptability in crossbred cattle. Asian-Australas J Anim Sci.

[6] West JW (2003). Effects of heat-stress on production in dairy cattle. J Dairy Sci.

[7] Gao S, Ma L, Zhou Z, Zhou Z, Baumgard LH, Jiang D (2019). Heat stress negatively affects the transcriptome related to overall metabolism and milk protein synthesis in mammary tissue of midlactating dairy cows. Physiol Genom.

[8] Baumgard LH, Rhoads RP (2013). Jr. Effects of heat stress on postabsorptive metabolism and energetics. Annu Rev Anim Biosci.

[9] Gao ST, Guo J, Quan SY, Nan XM, Fernandez MVS, Baumgard LH (2017). The effects of heat stress on protein metabolism in lactating Holstein cows. J Dairy Sci.

[10] Yue S, Wang Z, Wang L, Peng Q, Xue B (2020). Transcriptome functional analysis of mammary gland of cows in heat stress and thermoneutral condition. Animals.

[11] Dado-Senn B, Skibiel AL, Fabris TF, Zhang Y, Dahl GE, Peñagaricano F (2018). RNA-Seq reveals novel genes and pathways involved in bovine mammary involution during the dry period and under environmental heat stress. Sci Rep.

[12] Rawson P, Stockum C, Peng L, Manivannan B, Lehnert K, Ward HE (2012). Metabolic proteomics of the liver and mammary gland during lactation. J Proteom.

[13] Wheelock JB, Rhoads RP, VanBaale MJ, Sanders SR, Baumgard LH (2010). Effects of heat stress on energetic metabolism in lactating Holstein cows1. J Dairy Sci.

[14] Skibiel AL, Zachut M, do Amaral BC, Levin Y, Dahl GE (2018). Liver proteomic analysis of postpartum holstein cows exposed to heat stress or cooling conditions during the dry period. J Dairy Sci.

[15] Ma L, Yang Y, Zhao X, Wang F, Gao S, Bu D (2019). Heat stress induces proteomic changes in the liver and mammary tissue of dairy cows independent of feed intake: an iTRAQ study. PLoS ONE.

[16] Martínez RS, Palladino RA, Banchero G, Fernández-Martín R, Nanni M, Juliano N (2021). Providing heat-stress abatement to late-lactation holstein cows affects hormones, metabolite blood profiles, and hepatic gene expression but not productive responses. Appl Anim Sci.

[17] Li Y, Kong L, Deng M, Lian Z, Han Y, Sun B et al. Heat stress-responsive transcriptome analysis in the liver tissue of Hu Sheep. Genes (Basel). 2019;10(5). 10.3390/genes10050395 PMC6562622 31121974

[18] Bu D, Bionaz M, Wang M, Nan X, Ma L, Wang J (2017). Transcriptome difference and potential crosstalk between liver and mammary tissue in mid-lactation primiparous dairy cows. PLoS ONE.

[19] Fontoura ABP, Javaid A, De La Sáinz V, Salandy NS, Fubini SL, Grilli E et al. Heat stress develops with increased total-tract gut permeability, and dietary organic acid and pure botanical supplementation partly restores lactation performance in Holstein dairy cows. J Dairy Sci. 2022. 10.3168/jds.2022-21820 35931486

[20] Kendall PE, Tucker CB, Dalley DE, Clark DA, Webster JR (2008). Milking frequency affects the circadian body temperature rhythm in dairy cows. Livest Sci.

[21] Rico JE, Mathews AT, Lovett J, Haughey NJ, McFadden JW (2016). Palmitic acid feeding increases ceramide supply in association with increased milk yield, circulating nonesterified fatty acids, and adipose tissue responsiveness to a glucose challenge. J Dairy Sci.

[22] Chen S, Zhou Y, Chen Y, Gu J (2018). Fastp: an ultra-fast all-in-one FASTQ preprocessor. Bioinformatics.

[23] Dobin A, Davis CA, Schlesinger F, Drenkow J, Zaleski C, Jha S (2013). STAR: ultrafast universal RNA-seq aligner. Bioinformatics.

[24] Liao Y, Smyth GK, Shi W (2014). featureCounts: an efficient general purpose program for assigning sequence reads to genomic features. Bioinformatics.

[25] Mandric I, Temate-Tiagueu Y, Shcheglova T, Al Seesi S, Zelikovsky A, Măndoiu II (2017). Fast bootstrapping-based estimation of confidence intervals of expression levels and differential expression from RNA-Seq data. Bioinformatics.

[26] Love MI, Huber W, Anders S (2014). Moderated estimation of fold change and dispersion for RNA-seq data with DESeq2. Genome Biol.

[27] Gu Z, Eils R, Schlesner M (2016). Complex heatmaps reveal patterns and correlations in multidimensional genomic data. Bioinformatics.

[28] Kanehisa M, Goto S (2000). KEGG: kyoto encyclopedia of genes and genomes. Nucleic Acids Res.

[29] Wu T, Hu E, Xu S, Chen M, Guo P, Dai Z (2021). clusterProfiler 4.0: a universal enrichment tool for interpreting omics data. The Innovation.

[30] Livak KJ, Schmittgen TD (2001). Analysis of relative gene expression data using real-time quantitative PCR and the 2 – ∆∆CT method. Methods.

[31] Hu H, Miao Y-R, Jia L-H, Yu Q-Y, Zhang Q, Guo A-Y (2018). AnimalTFDB 3.0: a comprehensive resource for annotation and prediction of animal transcription factors. Nucleic Acids Res.

[32] Collier RJ, Dahl GE, VanBaale MJ (2006). Major advances Associated with Environmental Effects on dairy cattle. J Dairy Sci.

[33] Johnson HD. Environmental physiology and shelter engineering with special reference to domestic animals. LXVI, Temperature-humidity effects including influence of acclimation in feed and water consumption of Holstein cattle. 1963.

[34] Fuquay J (1981). Heat stress as it affects animal production. J Anim Sci.

[35] Beede D, Collier R (1986). Potential nutritional strategies for intensively managed cattle during thermal stress. J Anim Sci.

[36] Cowley F, Barber D, Houlihan A, Poppi D (2015). Immediate and residual effects of heat stress and restricted intake on milk protein and casein composition and energy metabolism. J Dairy Sci.

[37] Hartl FU (1996). Molecular chaperones in cellular protein folding. Nature.

[38] Arndt V, Rogon C, Höhfeld J (2007). To be, or not to be — molecular chaperones in protein degradation. Cell Mol Life Sci.

[39] Kon M, Cuervo AM (2010). Chaperone-mediated autophagy in health and disease. FEBS Lett.

[40] Milarski KL, Morimoto RI (1989). Mutational analysis of the human HSP70 protein: distinct domains for nucleolar localization and adenosine triphosphate binding. J Cell Biol.

[41] Bhat S, Kumar P, Kashyap N, Deshmukh B, Dige MS, Bhushan B (2016). Effect of heat shock protein 70 polymorphism on thermotolerance in Tharparkar cattle. Veterinary world.

[42] Tao S, Orellana RM, Weng X, Marins TN, Dahl GE, Bernard JK (2018). Symposium review: the influences of heat stress on bovine mammary gland function. J Dairy Sci.

[43] Kishore A, Sodhi M, Kumari P, Mohanty A, Sadana D, Kapila N (2014). Peripheral blood mononuclear cells: a potential cellular system to understand differential heat shock response across native cattle (Bos indicus), exotic cattle (Bos taurus), and riverine buffaloes (Bubalus bubalis) of India. Cell Stress Chaperones.

[44] Dangi SS, Gupta M, Nagar V, Yadav VP, Dangi SK, Shankar O (2014). Impact of short-term heat stress on physiological responses and expression profile of HSPs in Barbari goats. Int J Biometeorol.

[45] Romero RD, Montero Pardo A, Montaldo HH, Rodríguez AD, Hernández Cerón J (2013). Differences in body temperature, cell viability, and HSP-70 concentrations between Pelibuey and Suffolk sheep under heat stress. Trop Anim Health Prod.

[46] Baena MM, Tizioto PC, Meirelles SLC, Regitano LCDA. HSF1 and HSPA6 as functional candidate genes associated with heat tolerance in Angus cattle. Revista Brasileira de Zootecnia. 2018;47(0).

[47] Hageman J, Rujano MA, Van Waarde MA, Kakkar V, Dirks RP, Govorukhina N (2010). A DNAJB chaperone subfamily with HDAC-dependent activities suppresses toxic protein aggregation. Mol Cell.

[48] Rao R, Fiskus W, Ganguly S, Kambhampati S, Bhalla KN. Chapter seven - HDAC inhibitors and chaperone function. In: Grant S, editor. Advances in Cancer Research. Volume 116. Academic Press; 2012. pp. 239–62. 10.1016/B978-0-12-394387-3.00007-0 23088873

[49] Zhao R, Houry WA, Csermely P, Vígh L (2007). Molecular Interaction Network of the Hsp90 chaperone system. Molecular aspects of the stress response: chaperones, membranes and networks.

[50] Panaretou B, Siligardi G, Meyer P, Maloney A, Sullivan JK, Singh S (2002). Activation of the ATPase activity of hsp90 by the stress-regulated cochaperone aha1. Mol Cell.

[51] Lotz GP, Lin H, Harst A, Obermann WM (2003). Aha1 binds to the middle domain of Hsp90, contributes to client protein activation, and stimulates the ATPase activity of the molecular chaperone. J Biol Chem.

[52] Sonna LA, Fujita J, Gaffin SL, Lilly CM (2002). Invited review: effects of heat and cold stress on mammalian gene expression. J Appl Physiol.

[53] Pearce S, Mani V, Weber T, Rhoads R, Patience J, Baumgard L (2013). Heat stress and reduced plane of nutrition decreases intestinal integrity and function in pigs. J Anim Sci.

[54] Liu DY, He SJ, Liu SQ, Tang YG, Jin EH, Chen HL (2014). Daidzein enhances immune function in late lactation cows under heat stress. Anim Sci J.

[55] Cohen-Sfady M, Pevsner-Fischer M, Margalit R, Cohen IR (2009). Heat shock protein 60, via MyD88 Innate Signaling, protects B cells from apoptosis, spontaneous and Induced. J Immunol.

[56] He S, Yu Q, He Y, Hu R, Xia S, He J (2019). Dietary resveratrol supplementation inhibits heat stress-induced high-activated innate immunity and inflammatory response in spleen of yellow-feather broilers. Poult Sci.

[57] Ng IHW, Yeap YYC, Ong LSR, Jans DA, Bogoyevitch MA (2014). Oxidative stress impairs multiple regulatory events to drive persistent cytokine-stimulated STAT3 phosphorylation. Biochimica et Biophysica Acta (BBA). Mol Cell Res.

[58] Tao Z, Cheng M, Wang SC, Lv W, Hu HQ, Li CF (2015). JAK2/STAT3 pathway mediating inflammatory responses in heatstroke-induced rats. Int J Clin Exp Pathol.

[59] Horadagoda NU, Knox KM, Gibbs HA, Reid SW, Horadagoda A, Edwards SE (1999). Acute phase proteins in cattle: discrimination between acute and chronic inflammation. Vet Rec.

[60] Rawat M, Nighot M, Al-Sadi R, Gupta Y, Viszwapriya D, Yochum G (2020). IL1B increases intestinal tight Junction permeability by Up-regulation of MIR200C-3p, which degrades occludin mRNA. Gastroenterology.

[61] Marco-Ramell A, Arroyo L, Saco Y, García-Heredia A, Camps J, Fina M (2012). Proteomic analysis reveals oxidative stress response as the main adaptative physiological mechanism in cows under different production systems. J Proteom.

[62] Cui Y, Hao Y, Li J, Bao W, Li G, Gao Y (2016). Chronic heat stress induces immune response, oxidative stress response, and apoptosis of finishing pig liver: a proteomic approach. Int J Mol Sci.

[63] Hadsell DL, Olea W, Wei J, Fiorotto ML, Matsunami RK, Engler DA (2011). Developmental regulation of mitochondrial biogenesis and function in the mouse mammary gland during a prolonged lactation cycle. Physiol Genom.

[64] Laubenthal L, Hoelker M, Frahm J, Dänicke S, Gerlach K, Südekum K-H (2016). Mitochondrial DNA copy number and biogenesis in different tissues of early-and late-lactating dairy cows. J Dairy Sci.

[65] Favorit V, Hood WR, Kavazis AN, Villamediana P, Yap KN, Parry HA (2021). Mitochondrial bioenergetics of Extramammary tissues in lactating dairy cattle. Animals.

[66] Willis W, Jackman M, Bizeau M, Pagliassotti M, Hazel J (2000). Hyperthermia impairs liver mitochondrial function in vitro. Am J Physiology-Regulatory Integr Comp Physiol.

[67] Lambert SA, Jolma A, Campitelli LF, Das PK, Yin Y, Albu M (2018). The human transcription factors. Cell.

[68] Patel N, Varghese J, Masaratana P, Latunde-Dada GO, Jacob M, Simpson RJ (2014). The transcription factor ATOH8 is regulated by erythropoietic activity and regulates HAMP transcription and cellular pSMAD1,5,8 levels. Br J Haematol.

[69] Sampieri L, Di Giusto P, Alvarez C (2019). CREB3 transcription factors: ER-Golgi stress transducers as Hubs for Cellular Homeostasis. Front Cell Dev Biol.

[70] Liu C, Zhou B, Meng M, Zhao W, Wang D, Yuan Y et al. FOXA3 induction under endoplasmic reticulum stress contributes to non-alcoholic fatty liver disease. J Hepatol. 2021;75. 10.1016/j.jhep.2021.01.042 33548387

[71] Trougakos IP, Gonos ES (2006). Regulation of clusterin/apolipoprotein J, a functional homologue to the small heat shock proteins, by oxidative stress in ageing and age-related diseases. Free Radic Res.

[72] Bensellam M, Montgomery MK, Luzuriaga J, Chan JY, Laybutt DR (2015). Inhibitor of differentiation proteins protect against oxidative stress by regulating the antioxidant–mitochondrial response in mouse beta cells. Diabetologia.

[73] Salati S, Genovese E, Carretta C, Zini R, Bartalucci N, Prudente Z (2019). Calreticulin Ins5 and Del52 mutations impair unfolded protein and oxidative stress responses in K562 cells expressing CALR mutants. Sci Rep.

[74] Ma Y, Khan MZ, Xiao J, Alugongo GM, Chen X, Chen T (2021). Genetic markers Associated with milk production traits in dairy cattle. Agriculture.

