# Supplementary material for: Transcriptomic regulations of heat stress response in the liver of lactating dairy cows
Source: BMC Genomics. 2023 Jul 20;24:410. doi: 10.1186/s12864-023-09484-1 (PMC10360291; doi:10.1186/s12864-023-09484-1)
Supplement: Supplementary file 1 — Supplementary Material 1 [file 12864_2023_9484_MOESM1_ESM.docx]

**Table S1**

| Gene | GeneBank ID | Primer Sequence (5’-3’) | Product Size |
| --- | --- | --- | --- |
| PRAP1 | XM_005225715.4 | F: GGCCTGACATGAGAAGGCTCC  R: CCAGATGAGCTGGTTGTCCTT | 185 bp |
| ACACA | NM_174224.2 | F: GGAGACAAACAGGGACCATTAC  R: GTGGAAGGAATGCTTGGGAG | 187 bp |
| GPC3 | NM_001035463.2 | F: AGTGCTTTGCCTGGCTACAT  R: TTCCATTCCTCGCTGCCTTT | 115 bp |
| MIOX | NM_001101065.1 | F: TCCGAAACTACACGTCTGGC  R: ATAGGAGAAGCCCCCGAACT | 122 bp |
| DIO1 | NM_001122593.2 | F: CAACAGACCCTTGGTGCTGA  R: TCTTAAAAGCCCAGCCATCTGA | 161 bp |
| CRYAB | NM_174290.2 | F: ACATGAAGAGCGCCAGGATGA  R: GAGGCCTGTTTCCTTGGTCC | 150 bp |
| WFDC2 | NM_001076490.2 | F: GTCACAGGCTCAATCGCAGT  R: ACAGTCCTCATCCAAGACGC | 93 bp |
| HSPA1A | NM_203322.3 | F: TACGTGGCCTTCACCGATAC  R: GTCGTTGATGACGCGGAAAG | 171 bp |
| HSPB1 | NM_001025569.1 | F: CGGACGCACCCAGACCAGCCAGCAT  R: GGCTGTGGGCCGGATACCAGTCGCG | 105 bp |

**Table S2**

| Sample | Raw reads | Clean reads | Error (%) | Q20 (%) | Q30 (%) | GC (%) | Unique mapping rate (%) |
| --- | --- | --- | --- | --- | --- | --- | --- |
| HS_1 | 39896096 | 39667262 | 0.03 | 97.58 | 93.22 | 49.43 | 94.66 |
| HS_2 | 54990034 | 54622968 | 0.03 | 97.51 | 93.14 | 48.84 | 94.66 |
| HS_3 | 44358452 | 44127078 | 0.03 | 97.67 | 93.42 | 49.44 | 95.08 |
| PF_1 | 44179140 | 43932462 | 0.03 | 97.69 | 93.46 | 48.87 | 95.69 |
| PF_2 | 43048052 | 42791938 | 0.03 | 97.04 | 92.2 | 46.39 | 94.93 |
| PF_3 | 47116986 | 46839040 | 0.03 | 97.62 | 93.36 | 49.17 | 94.66 |
| TN_1 | 43244458 | 42989316 | 0.03 | 97.65 | 93.38 | 47.16 | 95.45 |
| TN_2 | 42745196 | 42467336 | 0.03 | 97.53 | 93.12 | 47.45 | 96.1 |
| TN_3 | 39950380 | 39726836 | 0.03 | 97.72 | 93.56 | 47.69 | 94.95 |
