# Supplementary figures and images for: Transcriptomic regulations of heat stress response in the liver of lactating dairy cows
Source: BMC Genomics. 2023 Jul 20;24:410. doi: 10.1186/s12864-023-09484-1 (PMC10360291; doi:10.1186/s12864-023-09484-1)

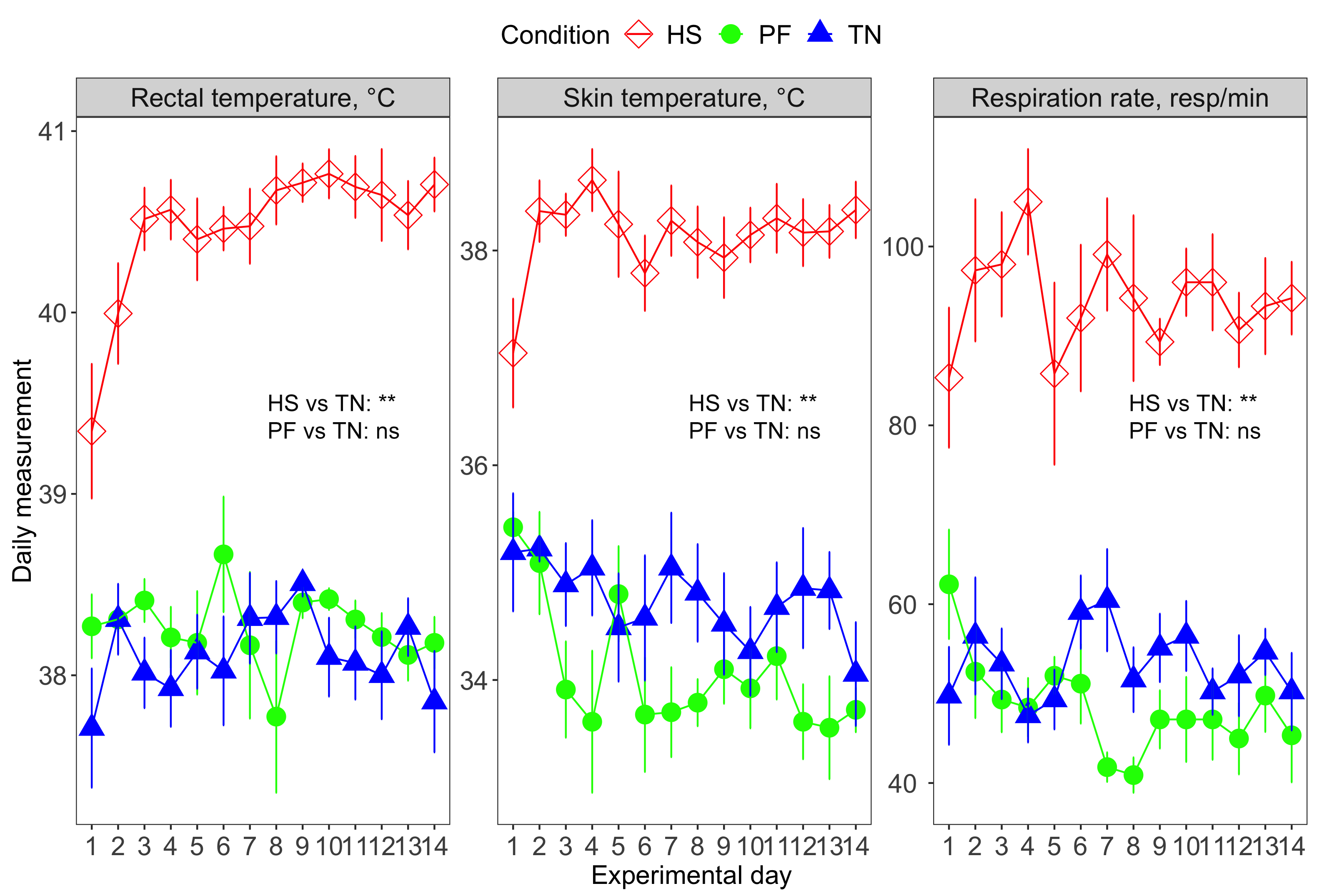

Supplement: Supplementary file 4 — Supplementary Material 4 [file 12864_2023_9484_MOESM4_ESM.tiff]

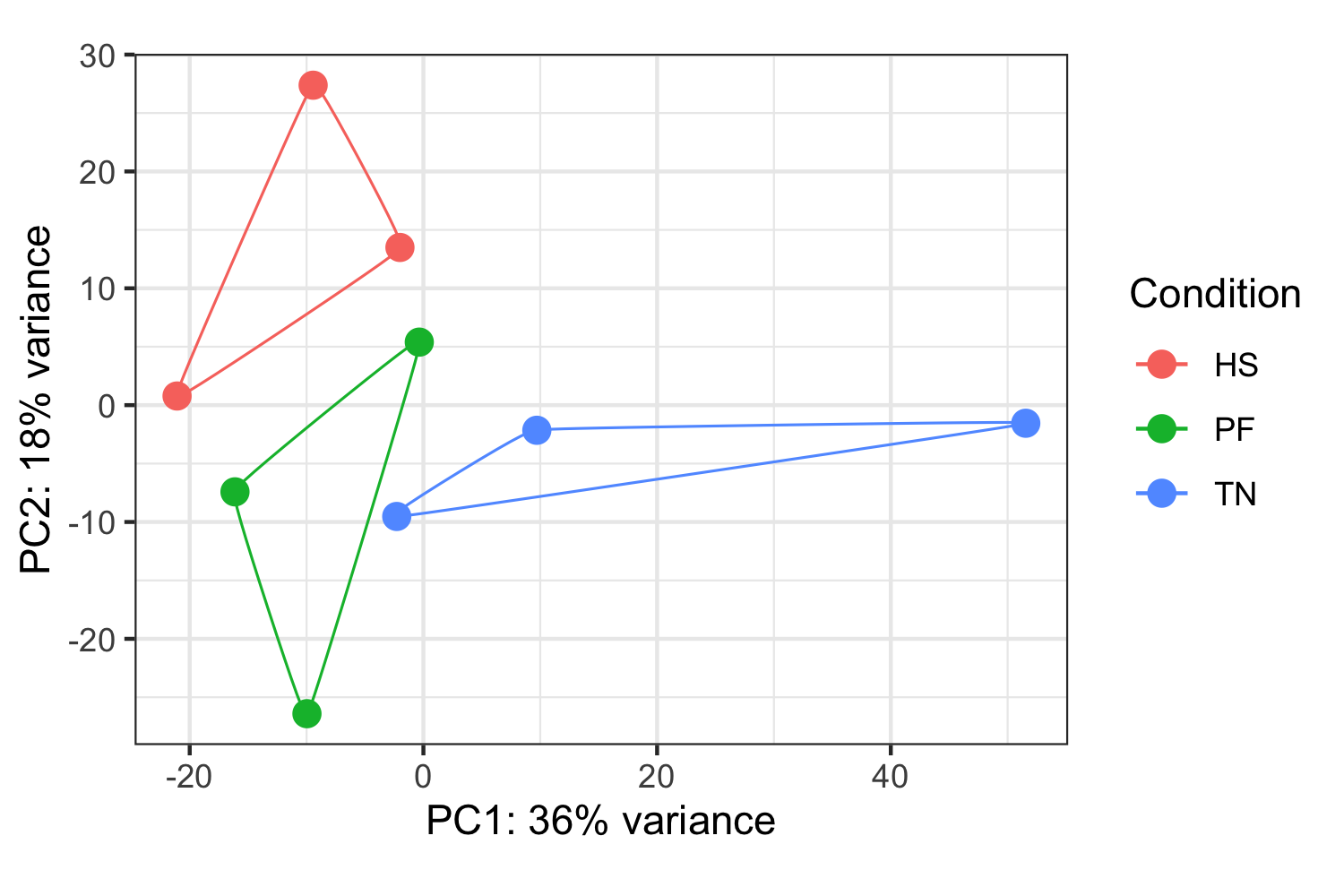

Supplement: Supplementary file 5 — Supplementary Material 5 [file 12864_2023_9484_MOESM5_ESM.tiff]

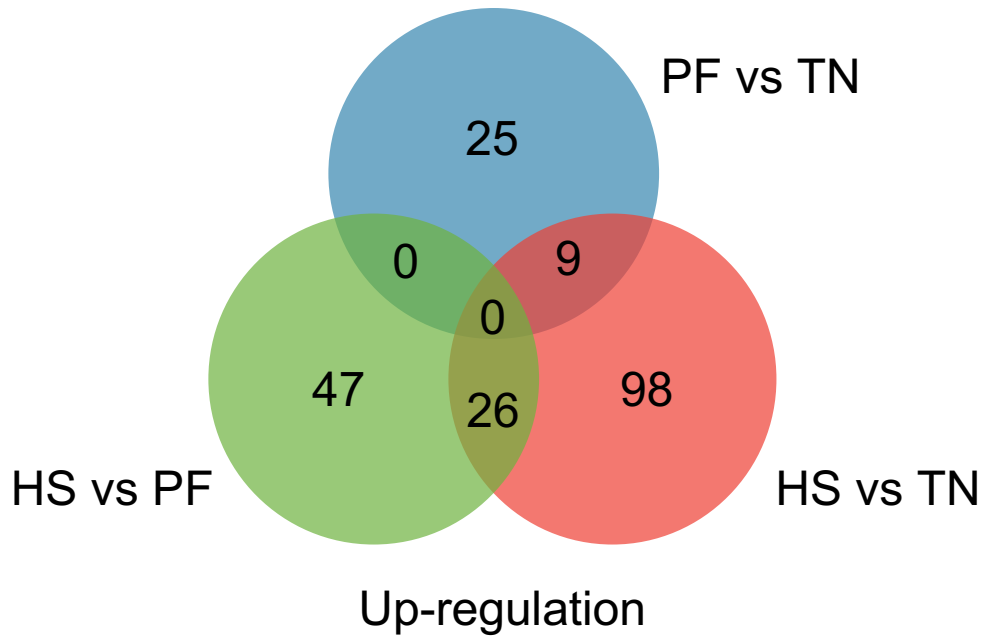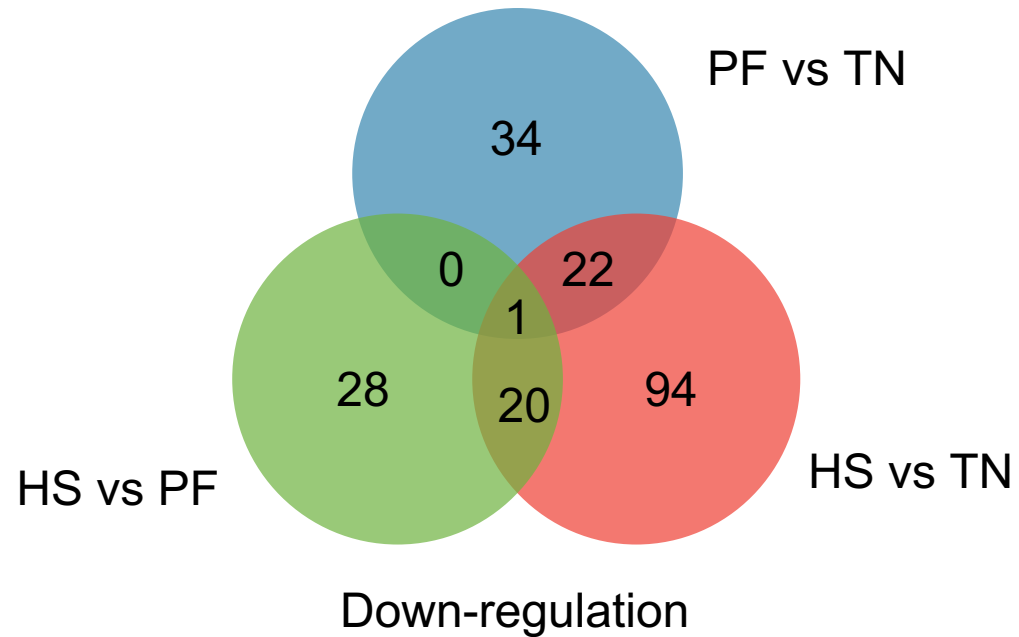

Supplement: Supplementary file 6 — Supplementary Material 6 [file 12864_2023_9484_MOESM6_ESM.pdf]

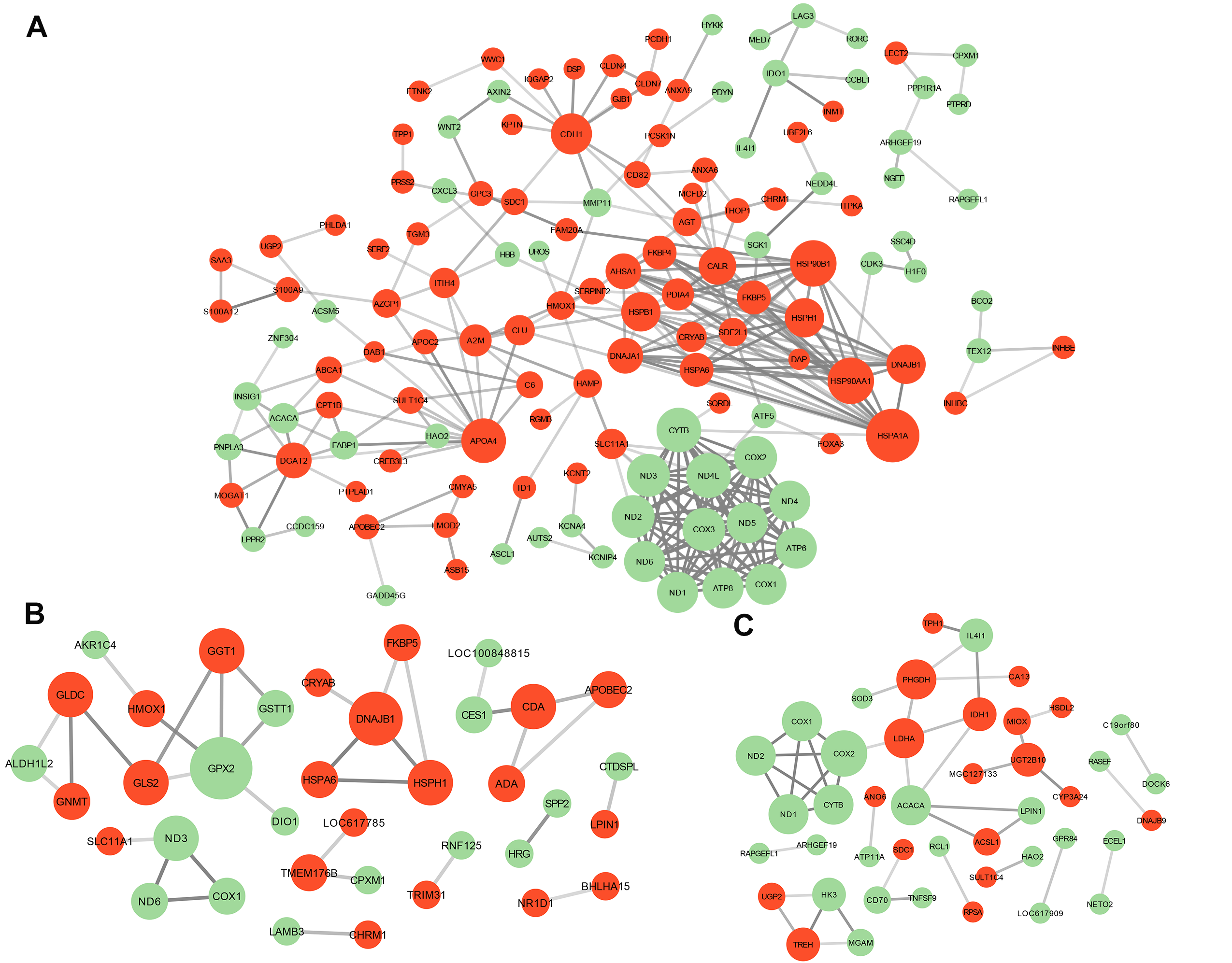

Supplement: Supplementary file 8 — Supplementary Material 8 [file 12864_2023_9484_MOESM8_ESM.tif]
