# Supplementary material for: Transcriptomic regulations of heat stress response in the liver of lactating dairy cows
Source: BMC Genomics. 2023 Jul 20;24:410. doi: 10.1186/s12864-023-09484-1 (PMC10360291; doi:10.1186/s12864-023-09484-1)
Supplement: Supplementary file 9 — Supplementary Material 9 [file 12864_2023_9484_MOESM9_ESM.docx]

**Supplementary information**

**Additional file 1: Table S1:** Primer list of selected genes for RT-qPCR validation. **Table S2:** Summary statistics of RNA-seq data. Unique mapping rate is obtained from STAR alignment result.

**Additional file 2**: **Table S3:** The gene list of differential expression analysis in pair-wise comparison. The FDR of the genes is obtained by DEseq2 R package.

**Additional file 3: Table S4:** The enrichment results of GO and KEGG for the DEGs.

**Additional file 4: Figure S1:** Measurement of clinical signs of heat stress response in cows under different conditions. **: p < 0.05; ns: no significance. TN: thermoneutrality; HS: heat stress; PF: pair-fed.

**Additional file 5: Figure S2:** PCA analysis of liver transcriptome in three conditions.

**Additional file 6: Figure S3:** Venn diagram of overlapping DEGs from pair-wise comparisons

**Additional file 7: Figure S4:** A: The GO enrichment of upregulated DEGs under PF; B: The KEGG enrichment of upregulated DEGs under PF; C: The GO enrichment of downregulated DEGs under PF; D: The KEGG enrichment of downregulated DEGs under PF. The brown node shows the enriched terms, and the size of brown node indicates the associated genes. The red or blue node shows the individual genes within each term, and the gradient of the gene color indicates the value of log2(Fold change).

**Additional file 8: Figure S5:** PPI networks of DEGs in pair wise comparison. A: HS vs TN; B: HS vs PF; C: PF vs TN. Red node: upregulated genes. Green node: downregulated genes. The color of the edge indicates the interaction score and the node size indicates the number of interactions with other proteins.
